# Supplementary material for: Health system interventions for adults with type 2 diabetes in low- and middle-income countries: A systematic review and meta-analysis
Source: PLoS Med. 2020 Nov 12;17(11):e1003434. doi: 10.1371/journal.pmed.1003434 (PMC7660583; doi:10.1371/journal.pmed.1003434)
Supplement: S1 Appendix — (PDF) [file pmed.1003434.s001.pdf]

S1 Appendix: PROSPERO registration and final review protocol

This supplementary file contains our PROSPERO registration (CRD42018106765) and most recent review protocol.

Please note that our final review protocol revision was completed on January 3, 2020. The PROSPERO website has experienced substantial delays in 2020. At the date of our manuscript's submission on May 5, 2020, our revision on the PROSPERO website is still listed as "Records that are being assessed." We include full documentation here for transparency.

You have 1 records

Records that are being assessed

These records have been submitted for publication and are being assessed by the editorial team. You cannot make changes to these records while they are going through the editorial process.

ID	Title	Status	Last edited
CRD42018106765	Health system interventions to improve clinical and patient-centered outcomes for adults with type 2 diabetes in low- and middle-income countries: A systematic review and meta-analysis	Registered	03/01/2020

Systematic review

1. * Review title.

Give the working title of the review, for example the one used for obtaining funding. Ideally the title should state succinctly the interventions or exposures being reviewed and the associated health or social problems. Where appropriate, the title should use the PI(E)COS structure to contain information on the Participants, Intervention (or Exposure) and Comparison groups, the Outcomes to be measured and Study designs to be included.

Health system interventions to improve clinical and patient-centered outcomes for adults with type 2 diabetes in low- and middle-income countries: A systematic review and meta-analysis

2. Original language title.

For reviews in languages other than English, this field should be used to enter the title in the language of the review. This will be displayed together with the English language title.

3. * Anticipated or actual start date.

Give the date when the systematic review commenced, or is expected to commence.

25/08/2018

4. * Anticipated completion date.

Give the date by which the review is expected to be completed.

01/03/2020

5. * Stage of review at time of this submission.

Indicate the stage of progress of the review by ticking the relevant Started and Completed boxes. Additional information may be added in the free text box provided.
Please note: Reviews that have progressed beyond the point of completing data extraction at the time of initial registration are not eligible for inclusion in PROSPERO. Should evidence of incorrect status and/or completion date being supplied at the time of submission come to light, the content of the PROSPERO record will be removed leaving only the title and named contact details and a statement that inaccuracies in the stage of the review date had been identified.
This field should be updated when any amendments are made to a published record and on completion and publication of the review. If this field was pre-populated from the initial screening questions then you are not able to edit it until the record is published.

The review has not yet started: No

Review stage	Started	Completed
Preliminary searches	Yes	Yes
Piloting of the study selection process	Yes	Yes
Formal screening of search results against eligibility criteria	Yes	Yes
Data extraction	Yes	No
Risk of bias (quality) assessment	Yes	Yes
Data analysis	Yes	No

Provide any other relevant information about the stage of the review here (e.g. Funded proposal, protocol not yet finalised).

6. * Named contact.

The named contact acts as the guarantor for the accuracy of the information presented in the register record.

David Flood

Email salutation (e.g. "Dr Smith" or "Joanne") for correspondence:

David

7. * Named contact email.

Give the electronic mail address of the named contact.

david@wuqukawoq.org

8. Named contact address

Give the full postal address for the named contact.

2 calle 5-43 zona 1, Santiago Sacatepéquez, Guatemala 03006

9. Named contact phone number.

Give the telephone number for the named contact, including international dialling code.

502-7840-3112

10. * Organisational affiliation of the review.

Full title of the organisational affiliations for this review and website address if available. This field may be completed as 'None' if the review is not affiliated to any organisation.

Wuqu’ Kawoq | Maya Health Alliance

Organisation web address:

http://www.wuqukawoq.org

11. * Review team members and their organisational affiliations.

Give the personal details and the organisational affiliations of each member of the review team. Affiliation refers to groups or organisations to which review team members belong. **NOTE: email and country are now mandatory fields for each person.**

David Flood. Wuqu’ Kawoq | Maya Health Alliance, Santiago Sacatepéquez, Guatemala; National Clinicians Scholars Program and Division of Hospital Medicine, University of Michigan
Jessica Gaulter. Medicine-Pediatrics Residency Program, University of Minnesota
Matthew Dunn. School of Public Health, University of Michigan
Sarah Jane Brown. Health Sciences Libraries, University of Minnesota, Minneapolis
Bradley H. Wagenaar. Department of Global Health, University of Washington
Elizabeth A. Rogers. Division of General Internal Medicine, University of Minnesota
Michele Heisler. Department of Internal Medicine and Institute for Healthcare Policy and Innovation, University of Michigan; Center for Clinical Management Research, Veterans Affairs Ann Arbor Healthcare System
Vineet Chopra. Division of Hospital Medicine, Department of Medicine, University of Michigan
Peter Rohloff. Centro de Investigación en La Salud Indígena, Wuqu’ Kawoq | Maya Health Alliance, Santiago Sacatepéquez, Guatemala; Division of Global Health Equity, Brigham and Women’s Hospital, Boston, MA, USA

12. * Funding sources/sponsors.

Give details of the individuals, organizations, groups or other legal entities who take responsibility for initiating, managing, sponsoring and/or financing the review. Include any unique identification numbers assigned to the review by the individuals or bodies listed.

The authors received no specific sources of funding for this work.

Grant number(s)

13. * Conflicts of interest.

List any conditions that could lead to actual or perceived undue influence on judgements concerning the main topic investigated in the review.

None

14. Collaborators.

Give the name and affiliation of any individuals or organisations who are working on the review but who are not listed as review team members. **NOTE: email and country are now mandatory fields for each person.**

Dr Timothy Wilt. Center for Chronic Disease Outcomes Research, Minneapolis Veterans Affairs Health Care System, Minneapolis, MN, USA

15. * Review question.

State the question(s) to be addressed by the review, clearly and precisely. Review questions may be specific or broad. It may be appropriate to break very broad questions down into a series of related more specific questions. Questions may be framed or refined using PI(E)COS where relevant.

What are the effectiveness and harms of health system interventions to improve glycemic control for adults with type 2 diabetes in in low- and middle-income countries?

16. * Searches.

State the sources that will be searched. Give the search dates, and any restrictions (e.g. language or publication period). Do NOT enter the full search strategy (it may be provided as a link or attachment.)

We will search the following electronic bibliographic databases: Ovid MEDLINE, Cochrane Library, EMBASE,

African Index Medicus, LILACS, and Global Index Medicus. We also will manually review reference lists of all included studies, related systematic reviews in PROSPERO, and the websites of major international diabetes organizations (International Diabetes Federation, World Diabetes Foundation, Global Alliance for Chronic Diseases, and World Health Organization) for additional potentially eligible studies.

Our search strategy will intersect three concepts: (1) type 2 diabetes, (2) LMICs, and (3) study design. We will not incorporate search terms relating to specific health system interventions as such interventions are summarized and indexed using widely heterogeneous descriptions.

17. URL to search strategy.

Give a link to a published pdf/word document detailing either the search strategy or an example of a search strategy for a specific database if available (including the keywords that will be used in the search strategies), or upload your search strategy.Do NOT provide links to your search results.

https://www.crd.york.ac.uk/PROSPEROFILES/106765_STRATEGY_20191120.pdf

Alternatively, upload your search strategy to CRD in pdf format. Please note that by doing so you are consenting to the file being made publicly accessible.

Do not make this file publicly available until the review is complete

18. * Condition or domain being studied.

Give a short description of the disease, condition or healthcare domain being studied. This could include health and wellbeing outcomes.

Type 2 diabetes conveys a high burden of death and disability globally that disproportionately impacts people in low- and middle-income countries (LMICs). Developing health systems interventions to address the morbidity, mortality, and costs related to diabetes in LMICs a thus a global health priority. However, systematic reviews examining the role of diabetes health systems or quality improvement strategies include few studies outside of high-income countries. This review aims to provide a general assessment of the effectiveness and harms of various health system interventions to improve type 2 diabetes outcomes in LMICs.

19. * Participants/population.

Give summary criteria for the participants or populations being studied by the review. The preferred format includes details of both inclusion and exclusion criteria.

Inclusion: Non-pregnant, community-dwelling adults aged 18 years or older with type 2 diabetes in low- or middle-income countries. Studies must include at least 100 total participants.

Exclusion: People with other forms of diabetes or diabetes risk (e.g., prediabetes, gestational diabetes, or type 1 diabetes). We also excluded interventions focused on Ramadan, insulin titration, or glucose self-monitoring as nuances related to these unique contexts limit generalizability.

20. * Intervention(s), exposure(s).

Give full and clear descriptions or definitions of the nature of the interventions or the exposures to be reviewed.

We define a health systems intervention as one “designed to improve professional practice and the delivery of effective health services.” Included studies describe an intervention within the four domains of EPOC health system interventions. We will exclude trials of patient behavior change alone if health-care professional behavior is not primarily influenced. We define “health-care professional” broadly to encompass physicians, nurses, pharmacists, and other allied health workers. Thus, an intervention aiming to improve glycemic control by training health-care professionals on diabetes education will be included; however, an intervention aiming to improve patient glycemic control solely through individualized diabetes education will be excluded.

21. * Comparator(s)/control.

Where relevant, give details of the alternatives against which the main subject/topic of the review will be compared (e.g. another intervention or a non-exposed control group). The preferred format includes details of both inclusion and exclusion criteria.

Usual or enhanced usual care

22. * Types of study to be included.

Give details of the types of study (study designs) eligible for inclusion in the review. If there are no restrictions on the types of study design eligible for inclusion, or certain study types are excluded, this should be stated. The preferred format includes details of both inclusion and exclusion criteria.

Included: We will include randomized trials and cluster randomized trials. Cluster randomized trials must have at least two intervention and control sites to be included.

Excluded: Non-randomized trials, controlled and uncontrolled before-after studies, cohort studies, cross-sectional studies, case-control studies, or case series.

23. Context.

Give summary details of the setting and other relevant characteristics which help define the inclusion or exclusion criteria.

We will include randomized controlled trials (RCTs) of health systems interventions conducted in at least one LMIC as defined by 2019 World Bank lending categories.We pre-specified that included studies were published in English, enrolled 100 or more participants for at least 24 weeks, and reported at least one of the following outcomes: glycemic changes, mortality, health-related quality of life, or resource utilization. We will exclude interventions focused on Ramadan, insulin titration, or glucose self-monitoring as nuances related to these unique contexts limit generalizability. No date restrictions were applied.

24. * Main outcome(s).

Give the pre-specified main (most important) outcomes of the review, including details of how the outcome is defined and measured and when these measurement are made, if these are part of the review inclusion criteria.

Outcomes: Glycemic changes (hemoglobin A1c or fasting blood glucose), mortality, health-related quality of

life (using a standardized instrument), or resource utilization (using cost-effectiveness or cost-utility analysis). Included studies had to report at least one of the above outcomes. If outcomes were not reported or missing, we will contact study authors via email to inquire about these data.

* Measures of effect

Please specify the effect measure(s) for you main outcome(s) e.g. relative risks, odds ratios, risk difference, and/or 'number needed to treat.

N/A

25. * Additional outcome(s).

List the pre-specified additional outcomes of the review, with a similar level of detail to that required for main outcomes. Where there are no additional outcomes please state 'None' or 'Not applicable' as appropriate to the review

None

* Measures of effect

Please specify the effect measure(s) for you additional outcome(s) e.g. relative risks, odds ratios, risk difference, and/or 'number needed to treat.

N/A

26. * Data extraction (selection and coding).

Describe how studies will be selected for inclusion. State what data will be extracted or obtained. State how this will be done and recorded.

Data management

An information specialist (SJB) will download all titles and abstracts retrieved by electronic searches to EndNote, remove duplicates, and then import to the web-based systematic review tool Covidence.

Selection process

Two review authors (DF and JG) will use Covidence to independently screen studies by title and abstract, and, subsequently, by full-text review. We will note the reason for ineligibility of studies selected for full-text screening but then subsequently excluded. We will resolve any disagreements in the screening process first by discussion between the two reviewers conducting the screening; if disagreements persist, a third review author will be consulted (PR). We will produce a PRISMA flow diagram of the selection process using Covidence. The unit of interest in this review is a single intervention, and we will aggregate multiple reports of the same intervention for extraction and analysis.

Data collection process

We will adapt relevant items from the standard EPOC data collection template to generate and pilot a customized electronic extraction spreadsheet. Three review authors (DF, JG, and MD) will each independently review all extracted data.

27. * Risk of bias (quality) assessment.

Describe the method of assessing risk of bias or quality assessment. State which characteristics of the studies will be assessed and any formal risk of bias tools that will be used.

Two reviewers (DF and JG) will independently assess risk of bias in Covidence for each study using the methodology recommended by EPOC. Disagreements will be resolved by discussion between the initial two reviews or, if required, with assistance from a third reviewer (PR).

28. * Strategy for data synthesis.

Provide details of the planned synthesis including a rationale for the methods selected. This **must not be generic text** but should be **specific to your review** and describe how the proposed analysis will be applied to your data.

We will generate tables describing included studies by EPOC taxonomy grouping, outcome, and World Bank income level. We will then conduct a narrative synthesis to summarize these results. We will conduct a meta-analysis for the outcome of glycemic changes (i.e., hemoglobin A1c) if we deem studies within similarly categorized groups to have sufficient clinical homogeneity. If a meta-analysis is conducted, we only will incorporate that are not judged as high risk of bias.

29. * Analysis of subgroups or subsets.

State any planned investigation of ‘subgroups’. Be clear and specific about which type of study or participant will be included in each group or covariate investigated. State the planned analytic approach.

As described above, we plan to synthesize and analyze data by EPOC taxonomy grouping, outcome, and World Bank income level.

30. * Type and method of review.

Select the type of review and the review method from the lists below. Select the health area(s) of interest for your review.

Type of review

Cost effectiveness
No

Diagnostic
No

Epidemiologic
No

Individual patient data (IPD) meta-analysis
No

Intervention
Yes

Meta-analysis
Yes

Methodology
No

Narrative synthesis
No

Network meta-analysis
No

Pre-clinical
No

Prevention
No

Prognostic
No

Prospective meta-analysis (PMA)
No

Review of reviews
No

Service delivery
No

Synthesis of qualitative studies
No

Systematic review
Yes

Other
No

Health area of the review

Alcohol/substance misuse/abuse
No

Blood and immune system
No

Cancer
No

Cardiovascular
Yes

Care of the elderly
No

Child health
No

Complementary therapies
No

Crime and justice
No

Dental
No

Digestive system
No

Ear, nose and throat
No

Education
No

Endocrine and metabolic disorders

Yes

Eye disorders
No

General interest
Yes

Genetics
No

Health inequalities/health equity
Yes

Infections and infestations
No

International development
No

Mental health and behavioural conditions
No

Musculoskeletal
No

Neurological
No

Nursing
No

Obstetrics and gynaecology
No

Oral health
No

Palliative care
No

Perioperative care
No

Physiotherapy
No

Pregnancy and childbirth
No

Public health (including social determinants of health)
No

Rehabilitation
No

Respiratory disorders
No

Service delivery
No

Skin disorders
No

Social care
No

Surgery
No

Tropical Medicine
No

Urological
No

Wounds, injuries and accidents
No

Violence and abuse
No

31. Language.

Select each language individually to add it to the list below, use the bin icon to remove any added in error.
English

There is not an English language summary

32. * Country.

Select the country in which the review is being carried out from the drop down list. For multi-national collaborations select all the countries involved.

Guatemala
United States of America

33. Other registration details.

Give the name of any organisation where the systematic review title or protocol is registered (such as with The Campbell Collaboration, or The Joanna Briggs Institute) together with any unique identification number assigned. (N.B. Registration details for Cochrane protocols will be automatically entered). If extracted data will be stored and made available through a repository such as the Systematic Review Data Repository (SRDR), details and a link should be included here. If none, leave blank.

34. Reference and/or URL for published protocol.

Give the citation and link for the published protocol, if there is one

Give the link to the published protocol.

https://www.crd.york.ac.uk/PROSPEROFILES/106765_PROTOCOL_20200103.pdf

Alternatively, upload your published protocol to CRD in pdf format. Please note that by doing so you are consenting to the file being made publicly accessible.

No I do not make this file publicly available until the review is complete

Please note that the information required in the PROSPERO registration form must be completed in full even if access to a protocol is given.

35. Dissemination plans.

Give brief details of plans for communicating essential messages from the review to the appropriate audiences.

Do you intend to publish the review on completion?

Yes

36. Keywords.

Give words or phrases that best describe the review. Separate keywords with a semicolon or new line. Keywords will help users find the review in the Register (the words do not appear in the public record but are included in searches). Be as specific and precise as possible. Avoid acronyms and abbreviations unless these are in wide use.

Diabetes Mellitus, Type 2; Global Health; Noncommunicable Diseases; Delivery of Health Care; Developing Countries; LMICs; LMIC; Low- and middle-income countries

37. Details of any existing review of the same topic by the same authors.

Give details of earlier versions of the systematic review if an update of an existing review is being registered, including full bibliographic reference if possible.

38. * Current review status.

Review status should be updated when the review is completed and when it is published. For newregistrations the review must be Ongoing.
Please provide anticipated publication date

Review_Ongoing

39. Any additional information.

Provide any other information the review team feel is relevant to the registration of the review.

40. Details of final report/publication(s).

This field should be left empty until details of the completed review are available.

Give the link to the published review.

Health system interventions to improve clinical and patient-centered outcomes for adults with type 2 diabetes in low- and middle-income countries: A systematic review and meta-analysis

Review protocol
Version: January 3, 2020

David Flood: Maya Health Alliance, Santiago Sacatepéquez, Guatemala; Division of Hospital Medicine, Department of Medicine, University of Michigan

Matthew Dunn: School of Public Health, University of Michigan

Jessica Gaulter: Medicine-Pediatrics Residency Program, University of Minnesota

Sarah Jane Brown: Health Sciences Libraries, University of Minnesota

Bradley H. Wagenaar: Department of Global Health, University of Washington

Elizabeth A. Rogers: Division of General Internal Medicine, University of Minnesota

Michele Heisler: Department of Internal Medicine and Institute for Healthcare Policy and Innovation, University of Michigan; Center for Clinical Management Research, Veterans Affairs Ann Arbor Healthcare System

Vineet Chopra: Division of Hospital Medicine, Department of Medicine, University of Michigan; Center for Clinical Management Research, Veterans Affairs Ann Arbor Healthcare System

Peter Rohloff: Maya Health Alliance, Santiago Sacatepéquez; Division of Global Health Equity, Brigham and Women's Hospital

Acknowledgements: Timothy J. Wilt: Center for Chronic Disease Outcomes Research, Minneapolis Veterans Affairs Health Care System, Minneapolis

Corresponding author:
David Flood
Wuqu' Kawoq | Maya Health Alliance
2 calle 5-43 zona 1
Santiago Sacatepéquez
Guatemala 03006

BACKGROUND

Rationale

Type 2 diabetes mellitus (T2DM) conveys a high burden of death and disability globally that disproportionately impacts people in low- and middle-income countries (LMICs). Of the 450 million people worldwide with T2DM, approximately 80% reside in LMICs.[1] From 1980-2014, the absolute number of people and percent of the population with diabetes increased more quickly in LMICs than in high-income countries (HICs).[2] People with diabetes in LMICs experience premature and morbidity and mortality relative to HICs.[3]

The design and implementation of interventions to effectively address the diabetes burden in LMICs is thus a global health priority. However, systematic reviews examining health systems interventions or quality improvement strategies for diabetes have included few studies in LMICs.[4-9] Given differences in health system infrastructure, resources, and population risk factors, it should not be assumed that interventions designed and tested in HICs are generalizable.

Among the few diabetes interventions reported from LMICs, studies have focused on prevention[10,11], regional evidence,[12] or specific interventions such as task shifting,[13,14] deployment of community health workers,[15-17] or lifestyle change.[18,19] Other reviews of cardiovascular disease interventions in LMICs have not emphasized diabetes.[20] To date, no review has synthesized evidence across different types of health system interventions to improve type 2 diabetes outcomes in LMICs.

Therefore, we will examine the impact of health system interventions aiming to improve clinical and patient-centered outcomes for adults with T2DM in LMICs.

METHODS

This systematic review will be conducted based on guidance from the Cochrane Effective Practice and Organisation of Care (EPOC) group, which conducts reviews on health systems interventions.[21] We registered the review in PROSPERO (CRD42018106765) and followed the Preferred Reporting Items for Systematic Reviews and Meta-analyses (PRISMA) guidelines.[22]

Framework

Our PICO summary and analytic framework is described below and in Figure 1. This framework is based on an example from the literature.[23,24]

- **Population**: Non-pregnant, community-dwelling adults with type 2 diabetes in low- or middle-income countries
- **Interventions**: Health systems interventions aiming to improve clinical and patient-centered outcomes
- **Comparator**: Usual Care
- **Outcomes**:

- Clinical outcomes: Mortality and health-related quality of life (using any standardized instrument)
- Intermediate outcomes: Absolute change in glycemic control indicator (hemoglobin A1c of fasting blood glucose)
- Resource utilization: Cost-effectiveness (using cost-effectiveness or cost-utility analysis)

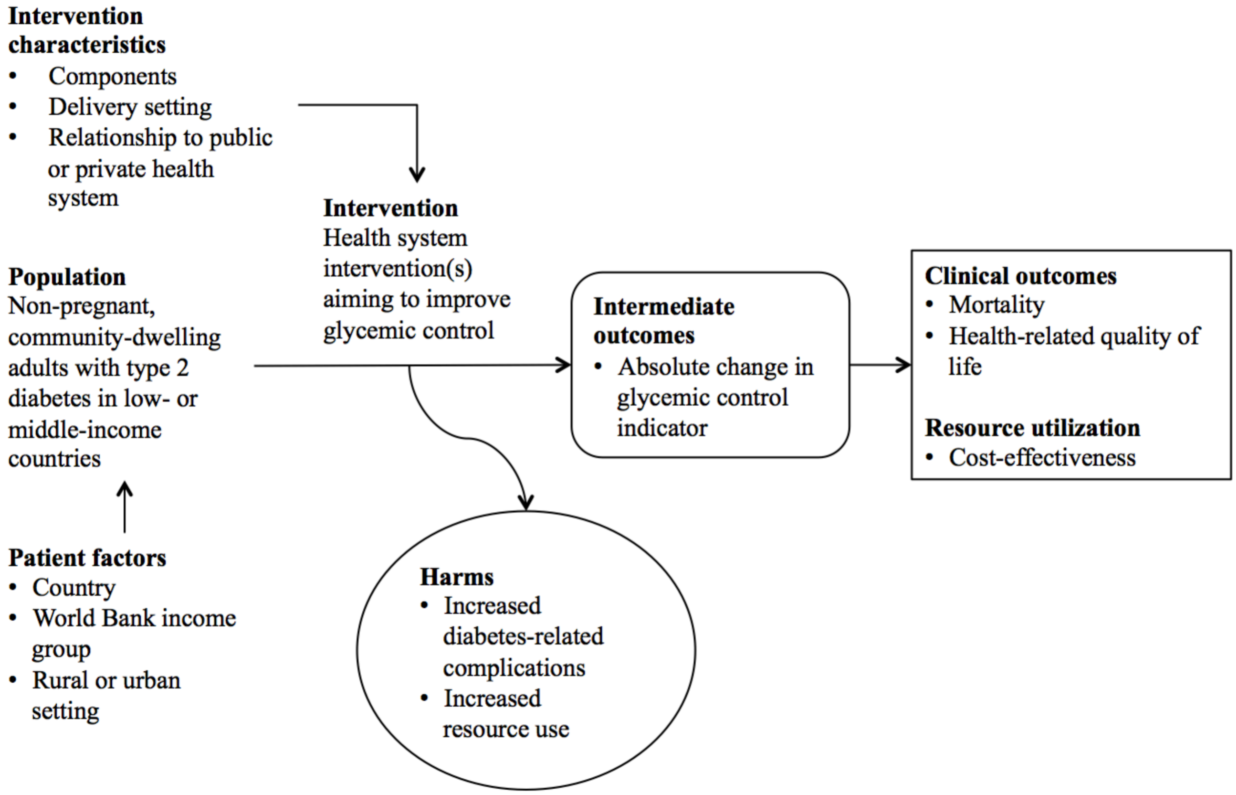

Figure 1: PICO framework.

Search strategy and selection criteria

Our search strategy intersects three concepts: type 2 diabetes, LMICs, and study design.[25,26] (See Figure 2: Schematic of search strategy.) With the assistance of a research librarian (SJB), we will perform serial searches of Ovid MEDLINE, Cochrane Library, EMBASE, African Index Medicus, LILACS, and Global Index Medicus. To identify additional potentially eligible studies, we also manually will review the references of included studies, related systematic reviews, and the websites of major international diabetes organizations. All searches will be developed in Ovid MEDLINE and syntax will be translated to other bibliographic databases. To ensure search quality, a second reference librarian will peer-review the search terms.

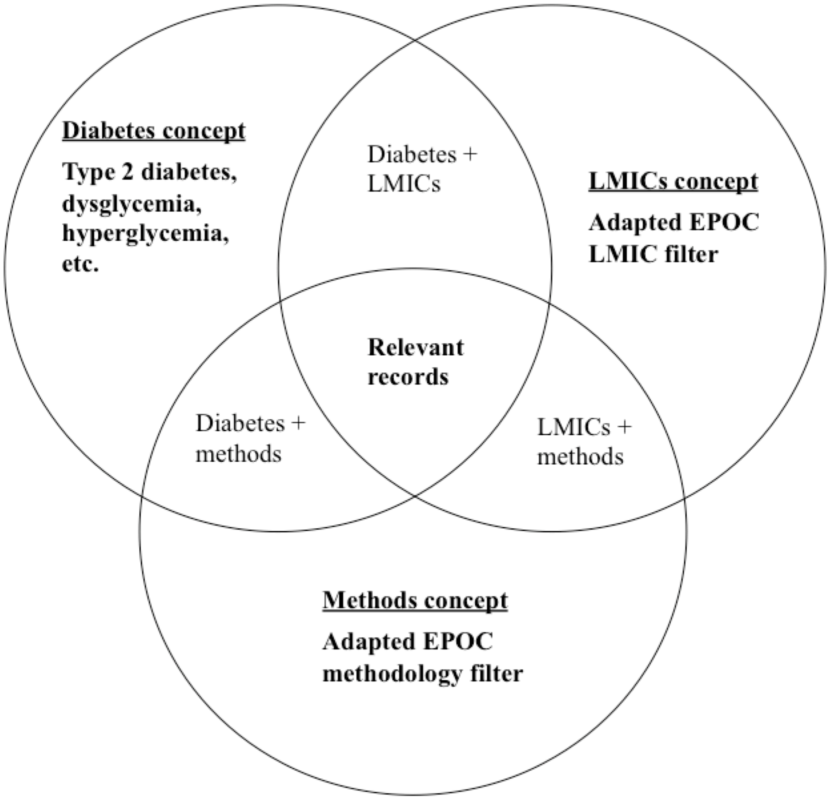

Figure 2: Schematic of search strategy.

We will included randomized controlled trials (RCTs) of health systems interventions conducted in at least one LMIC as defined by 2019 World Bank lending categories.[27] Both individual and cluster RCTs will be eligible. We pre-specified that included studies were published in English, enrolled 100 or more participants for at least 24 weeks, and reported at least one of the following outcomes: glycemic changes, mortality, health-related quality of life, or resource utilization. We will exclude interventions focused on Ramadan, insulin titration, or glucose self-monitoring as nuances related to these unique contexts limit generalizability. No date restrictions were applied.

We will defin a health systems intervention as one "designed to improve professional practice and the delivery of effective health services."[21] Included studies will describe an intervention within the four domains of EPOC health system interventions:[28]

- Delivery arrangements: Changes in how, when and where healthcare is organized and delivered, and who delivers healthcare.
- Financial arrangements: Changes in how funds are collected, insurance schemes, how services are purchased, and the use of targeted financial incentives or disincentives
- Governance arrangements: Rules or processes that affect the way in which powers are exercised, particularly with regard to authority, accountability, openness, participation, and coherence.

- Implementation strategies: Interventions designed to bring about changes in healthcare organizations, the behavior of healthcare professionals, or the use of health services by healthcare recipients.

We will exclude trials of patient behavior change alone if health-care professional behavior is not primarily influenced.[21] We define "health-care professional" broadly to encompass physicians, nurses, pharmacists, and other allied health workers. Thus, an intervention *training* health-care professionals on diabetes education will be included; however, an intervention aiming to improve outcomes solely through individualized diabetes education will be excluded.[21]

Data analysis

A reference librarian (SJB) will download all records to Endnote, remove duplicates, and import records to Covidence ®.[29] Two authors (DF and JG) will independently screen studies by title and abstract and, subsequently, by full-text review. Disagreements will be resolved first by consensus and, if needed, in consultation with a third author (PR). We will aggregate multiple reports of the same intervention for extraction and analysis. We will use the TIDieR checklist and EPOC template to structure extraction.[30,31] One author (DF) will extract summary data into a customized electronic spreadsheet, and a second author (JG) independently verified the extracted data. In addition relevant outcomes, we will extract study elements including country, setting, duration and follow-up, number of participants enrolled, intervention description, and comparator. We will classify each study by one or more taxonomy category from EPOC.[28] Within each category, we will use consensus between two reviewers (DF and JG) to group interventions into similar subcategories. If outcomes are not reported or missing, we will authors twice over four weeks via email to obtain these data. To assess risk of bias, two reviewers (DF and JG) independently will assess studies using the Cochrane EPOC tool.[32] Disagreements will be resolved by discussion between the two reviewers and, if needed, in consultation with a third reviewer (PR). We will summarize findings using EPOC guidance.[33]

Statistical analysis

We will conduct a meta-analysis if we deem studies within similarly categorized groups to have sufficient clinical homogeneity. We likely would conduct a meta-analysis for the glycemic outcome only and limit studies to those not judged as having high risk of bias. We plan to use random-effects meta-analysis models for mean between-group difference of hemoglobin A1c (HbA1c) change in each intervention category. The meta-analysis may be restricted to trials not classified as high risk of bias. Sample sizes for cluster RCTs would be adjusted to account for the design effect using the intracluster correlation coefficient.[34] We would follow the methodology recommended in the Cochrane handbook to derive within-group mean and standard deviation when complete HbA1c results were not reported.[34] Analyses will be performed in Stata (16.0). Heterogeneity will be quantified by calculating I^2 and T^2. We might perform exploratory analyses by excluding trials in which the comparator arm was enhanced usual care and/or constructing meta-regression models with baseline HbA1c as a covariate. Publication bias would be assessed by visual inspection of funnel plots. We would use GRADE and EPOC guidance to prepare a summary of findings table for the outcome of glycemic control.[35,36]

Role of the funding source

There is no funding source for this study. The corresponding author will have full access to all the data in the study and will have final responsibility for the decision to submit for publication.

References

1. Cho NH, Shaw JE, Karuranga S, Huang Y, da Rocha Fernandes JD, Ohlrogge AW, et al. IDF Diabetes Atlas: Global estimates of diabetes prevalence for 2017 and projections for 2045. Diabetes Res Clin Pract. 2018 Apr;138:271-81. doi:http://dx.doi.org/10.1016/j.diabres.2018.02.023 PMID: 29496507

2. Worldwide trends in diabetes since 1980: a pooled analysis of 751 population-based studies with 4.4 million participants. Lancet. 2016 Apr 09;387(10027):1513-30. doi:http://dx.doi.org/10.1016/s0140-6736(16)00618-8 PMID: 27061677

3. World Health Organization. Global report on diabetes. Geneva, Switzerland: World Health Organization; 2016.

4. Ong SE, Koh JJK, Toh SES, Chia KS, Balabanova D, McKee M, et al. Assessing the influence of health systems on Type 2 Diabetes Mellitus awareness, treatment, adherence, and control: A systematic review. PLoS One. 2018;13(3):e0195086. doi:http://dx.doi.org/10.1371/journal.pone.0195086 PMID: 29596495

5. Lim LL, Lau ESH, Kong APS, Davies MJ, Levitt NS, Eliasson B, et al. Aspects of Multicomponent Integrated Care Promote Sustained Improvement in Surrogate Clinical Outcomes: A Systematic Review and Meta-analysis. Diabetes Care. 2018 Jun;41(6):1312-20. doi:http://dx.doi.org/10.2337/dc17-2010 PMID: 29784698

6. Murphy ME, Byrne M, Galvin R, Boland F, Fahey T, Smith SM. Improving risk factor management for patients with poorly controlled type 2 diabetes: a systematic review of healthcare interventions in primary care and community settings. BMJ Open. 2017 Aug 4;7(8):e015135. doi:http://dx.doi.org/10.1136/bmjopen-2016-015135 PMID: 28780542

7. Renders CM, Valk GD, Griffin SJ, Wagner EH, Eijk Van JT, Assendelft WJ. Interventions to improve the management of diabetes in primary care, outpatient, and community settings: a systematic review. Diabetes Care. 2001 Oct;24(10):1821-33. PMID: 11574449

8. Tricco AC, Ivers NM, Grimshaw JM, Moher D, Turner L, Galipeau J, et al. Effectiveness of quality improvement strategies on the management of diabetes: a systematic review and meta-analysis. Lancet. 2012 Jun 16;379(9833):2252-61. doi:http://dx.doi.org/10.1016/s0140-6736(12)60480-2 PMID: 22683130

9. Terens N, Vecchi S, Bargagli AM, Agabiti N, Mitrova Z, Amato L, et al. Quality improvement strategies at primary care level to reduce inequalities in diabetes care: an equity-oriented systematic review. BMC Endocr Disord. 2018 May 29;18(1):31. doi:http://dx.doi.org/10.1186/s12902-018-0260-4 PMID: 29843692

10. Shirinzadeh M, Afshin-Pour B, Angeles R, Gaber J, Agarwal G. The effect of community-based programs on diabetes prevention in low- and middle-income countries: a systematic review and meta-analysis. Global Health. 2019 Feb 1;15(1):10. doi:http://dx.doi.org/10.1186/s12992-019-0451-4 PMID: 30709362

11. Gyawali B, Bloch J, Vaidya A, Kallestrup P. Community-based interventions for prevention of Type 2 diabetes in low- and middle-income countries: a systematic review. Health Promot Int. 2018 Oct 16. doi:http://dx.doi.org/10.1093/heapro/day081 PMID: 30329052

12. Kaselitz E, Rana GK, Heisler M. Public Policies and Interventions for Diabetes in Latin America: a Scoping Review. Curr Diab Rep. 2017 Aug;17(8):65. doi:http://dx.doi.org/10.1007/s11892-017-0888-x PMID: 28721593

13. Ogedegbe G, Gyamfi J, Plange-Rhule J, Surkis A, Rosenthal DM, Airhihenbuwa C, et al. Task shifting interventions for cardiovascular risk reduction in low-income and middle-income countries: a systematic review of randomised controlled trials. BMJ Open. 2014 Oct 16;4(10):e005983. doi:http://dx.doi.org/10.1136/bmjopen-2014-005983 PMID: 25324324

14. Joshi R, Alim M, Kengne AP, Jan S, Maulik PK, Peiris D, et al. Task shifting for non-communicable disease management in low and middle income countries--a systematic review. PLoS One. 2014;9(8):e103754. doi:http://dx.doi.org/10.1371/journal.pone.0103754 PMID: 25121789

15. Khetan AK, Purushothaman R, Chami T, Hejjaji V, Madan Mohan SK, Josephson RA, et al. The Effectiveness of Community Health Workers for CVD Prevention in LMIC. Glob Heart. 2017 Sep;12(3):233-43 e6. doi:http://dx.doi.org/10.1016/j.gheart.2016.07.001 PMID: 27993594

16. Jeet G, Thakur JS, Prinja S, Singh M. Community health workers for non-communicable diseases prevention and control in developing countries: Evidence and implications. PLoS One. 2017;12(7):e0180640. doi:http://dx.doi.org/10.1371/journal.pone.0180640 PMID: 28704405

17. Egbujie BA, Delobelle PA, Levitt N, Puoane T, Sanders D, van Wyk B. Role of community health workers in type 2 diabetes mellitus self-management: A scoping review. PLoS One. 2018;13(6):e0198424. doi:http://dx.doi.org/10.1371/journal.pone.0198424 PMID: 29856846

18. Hearn J, Ssinabulya I, Schwartz JI, Akiteng AR, Ross HJ, Cafazzo JA. Self-management of non-communicable diseases in low- and middle-income countries: A scoping review. PLoS One. 2019;14(7):e0219141. doi:http://dx.doi.org/10.1371/journal.pone.0219141 PMID: 31269070

19. Afable A, Karingula NS. Evidence based review of type 2 diabetes prevention and management in low and middle income countries. World J Diabetes. 2016 May 25;7(10):209-29. doi:http://dx.doi.org/10.4239/wjd.v7.i10.209 PMID: 27226816

20. Lee ES, Vedanthan R, Jeemon P, Kamano JH, Kudesia P, Rajan V, et al. Quality Improvement for Cardiovascular Disease Care in Low- and Middle-Income Countries: A Systematic Review. PLoS One. 2016;11(6):e0157036. doi:http://dx.doi.org/10.1371/journal.pone.0157036 PMID: 27299563

21. EPOC. Scope of our work. Available from: https://epoc.cochrane.org/scope-our-work.

22. Moher D, Liberati A, Tetzlaff J, Altman DG. Preferred reporting items for systematic reviews and meta-analyses: the PRISMA statement. PLoS Med. 2009 Jul 21;6(7):e1000097. doi:http://dx.doi.org/10.1371/journal.pmed.1000097 PMID: 19621072

23. Greer N, Bolduc J, Geurkink E, Koeller E, Rector T, Olson K, et al. VA Evidence-based Synthesis Program Reports. Pharmacist-Led Chronic Disease Management: A Systematic Review of Effectiveness and Harms Compared to Usual Care. Washington (DC): Department of Veterans Affairs (US); 2015.

24. Greer N, Bolduc J, Geurkink E, Rector T, Olson K, Koeller E, et al. Pharmacist-led Chronic Disease Management: A Systematic Review of Effectiveness and Harms Compared With Usual Care. Ann Intern Med. 2016 Apr 26. doi:http://dx.doi.org/10.7326/m15-3058 PMID: 27111098

25. Effective Practice and Organisation of Care (EPOC). LMIC Filters. 2012; Available from: http://epoc.cochrane.org/lmic-filters.

26. Effective Practice and Organisation of Care (EPOC). How to develop a search strategy for an intervention review. 2017; Available from: http://epoc.cochrane.org/epoc-specific-resources-review-authors

27. World Bank. World Bank Country and Lending Groups. 2018.

28. Effective Practice and Organisation of Care (EPOC). EPOC taxonomy. 2015; Available from: http://epoc.cochrane.org/sites/epoc.cochrane.org/files/public/uploads/epoc_taxonomy_13.12.16.pdf.

29. Veritas Health Innovation. Covidence systematic review software. Melbourne, Australia.

30. Effective Practice and Organisation of Care (EPOC). Data collection form 2017; Available from: http://epoc.cochrane.org/epoc-specific-resources-review-authors.

31. Hoffmann TC, Glasziou PP, Boutron I, Milne R, Perera R, Moher D, et al. Better reporting of interventions: template for intervention description and replication (TIDieR) checklist and guide. BMJ. 2014 Mar 7;348:g1687. doi:http://dx.doi.org/10.1136/bmj.g1687 PMID: 24609605

32. Effective Practice and Organisation of Care (EPOC). Suggested risk of bias criteria for EPOC reviews. 2017; Available from: http://epoc.cochrane.org/resources/epoc-resources-review-authors.

33. Effective Practice and Organisation of Care (EPOC). Synthesising results when it does not make sense to do a meta-analysis. 2017; Available from: http://epoc.cochrane.org/resources/epoc-resources-review-authors.

34. Higgins J, Green S, editors. Cochrane Handbook for Systematic Reviews of Interventions: The Cochrane Collaboration; 2011.

35. Guyatt GH, Oxman AD, Vist GE, Kunz R, Falck-Ytter Y, Alonso-Coello P, et al. GRADE: an emerging consensus on rating quality of evidence and strength of recommendations. BMJ. 2008 Apr 26;336(7650):924-6. doi:http://dx.doi.org/10.1136/bmj.39489.470347.AD PMID: 18436948

36. Effective Practice and Organisation of Care (EPOC). EPOC worksheets for preparing a Summary of Findings (SoF) table using GRADE. 2017; Available from: http://epoc.cochrane.org/resources/epoc-resources-review-authors.
