# Supplementary material for: Health system interventions for adults with type 2 diabetes in low- and middle-income countries: A systematic review and meta-analysis
Source: PLoS Med. 2020 Nov 12;17(11):e1003434. doi: 10.1371/journal.pmed.1003434 (PMC7660583; doi:10.1371/journal.pmed.1003434)
Supplement: S3 Appendix — (PDF) [file pmed.1003434.s003.pdf]

S3 Appendix: Search strategy

Database(s): **Ovid MEDLINE(R) and Epub Ahead of Print, In-Process & Other Non-Indexed Citations, Daily, and**

#	Searches	Results
1	exp Developing Countries/	70246
2	(Africa or Asia or Caribbean or "West Indies" or "South America" or "Latin America" or "Central America").ti,ab,kf,cp.	191521
3	(Afghanistan or Albania or Algeria or Angola or Armenia or Azerbaijan or Bangladesh or Byelarus or Byelorussian or Belarus or Belorussian or Belorussia or Belize or Benin or Bhutan or Bolivia or Bosnia or Herzegovina or Hercegovina or Botswana or Brazil or Bulgaria or Burkina Faso or Burkina Fasso or Upper Volta or Burundi or Urundi or Cabo Verde or Cape Verde or Cambodia or Khmer Republic or Kampuchea or Cameroon or Cameroons or Cameron or Camerons or Central African Republic or Chad or China or Colombia or Comoros or Comoro Islands or Comores or Mayotte or Congo or Zaire or Costa Rica or Cote d'Ivoire or Ivory Coast or Cuba or Djibouti or French Somaliland or Dominica or Dominican Republic or Ecuador or Egypt or El Salvador or Equatorial Guinea or Eritrea or Ethiopia or Fiji or Gabon or Gabonese Republic or Gambia or Georgia Republic or Georgian Republic or Ghana or Grenada or Guatemala or Guinea or Guinea-Bissau or Guyana or Haiti or Honduras or India or Indonesia or Iran or Iraq or Jamaica or Jordan or Kazakhstan or Kazakh or Kenya or Kiribati or Kosovo or Kyrgyzstan or Kirghizia or Kyrgyz Republic or Kirghiz or Kirgizstan or Lao PDR or Laos or Lebanon or Lesotho or Basutoland or Liberia or Libya or Macedonia or Madagascar or Malagasy Republic or Malawi or Malaysia or Malaya or Malay or Sabah or Sarawak or Maldives or Mali or Nyasaland or Marshall Islands or Mauritania or Mauritius or Agalega Islands or Mexico or Micronesia or Moldova or Moldovia or Moldovian or Mongolia or Montenegro or Morocco or Ifni or Mozambique or Myanmar or Myanma or Burma or Namibia or Nauru or Nepal or Nicaragua or Niger or Nigeria or North Korea or Pakistan or Papua New Guinea or Paraguay or Peru or Philippines or Philipines or Phillipines or Phillippines or Romania or Rumania or Roumania or Russian Federation or Rwanda or Ruanda or Samoa or Samoan Islands or Navigator Island or Navigator Islands or Sao Tome or Senegal or Serbia or Sierra Leone or Solomon Islands or Somalia or South Africa or South Sudan or Sri Lanka or Ceylon or Saint Lucia or St Lucia or Saint Vincent or St Vincent or Grenadine* or Sudan or Surinam* or Swaziland or Syria* or Tajikistan or Tadzhikistan or Tadjikistan or Tadzhik or Tanzania or Thailand or Timor-Leste or Timor or Togo or Togolese or Tonga or Tunisia or Turkey or Turkmenistan or Turkmen or Tuvalu or Ellice Islands or Uganda or Ukraine or Uzbekistan or Uzbek or Vanuatu or New Hebrides or	2132679

	Venezuela or Vietnam or Viet Nam or West Bank or Gaza or Yemen* or Zambia or Zimbabwe).ti,ab,kf,cp.	
4	((developing or "less* developed" or "under developed" or underdeveloped or "middle income" or "low* income") adj3 (countr* or nation* or economy or economies)).ti,ab.	74874
5	(low* adj3 (gdp or gnp or "gross domestic" or "gross national")).ti,ab,kf.	413
6	((low adj3 countr*) or (middle adj3 countr*)).ti,ab,kf.	22002
7	(lmic or lmics or "third world" or lami countr*).ti,ab,kf.	5749
8	1 or 2 or 3 or 4 or 5 or 6 or 7	2269573
9	exp Diabetes Mellitus, Type 2/	115224
10	(type* adj3 ("2" or "II" or two*) adj3 (diabete* or diabetic*)).ti,ab,kf.	124740
11	((maturit* or adult* or slow*) adj3 onset* adj3 (diabete* or diabetic*)).ti,ab,kf.	2737
12	((ketosis-resistant* or stable*) adj3 (diabete* or diabetic*)).ti,ab,kf.	707
13	((non-insulin* or non insulin* or noninsulin*) adj3 depend* adj3 (diabete* or diabetic*)).ti,ab,kf.	11716
14	(MODY or NIDDM or T2DM or T2D).ti,ab,kf.	29875
15	9 or 10 or 11 or 12 or 13 or 14	169404
16	randomized controlled trial.pt.	464507
17	pragmatic clinical trial.pt.	814
18	multicenter study.pt.	236284
19	non-randomized controlled trials as topic/	368
20	controlled before-after studies/	336
21	(randomis* or randomiz* or randomly).ti,ab.	779659
22	(trial or cluster or multicenter or multi center or multicentre or multi centre).ti.	242680
23	(intervention? or controlled or control group? or quasiexperiment* or quasi experiment*).ti,ab.	1683425
24	16 or 17 or 18 or 19 or 20 or 21 or 22 or 23	2388519
25	exp Animals/	21648572
26	Humans/	17172017
27	25 not (25 and 26)	4476555
28	review.pt.	2403044
29	meta analysis.pt.	90229
30	news.pt.	190572
31	comment.pt.	723381
32	editorial.pt.	462671

33	comment on.cm.	723377
34	(systematic review or literature review).ti.	114475
35	or/27-34	7950632
36	24 not 35	1825269
37	8 and 15 and 36	3914
