# Supplementary material for: Health system interventions for adults with type 2 diabetes in low- and middle-income countries: A systematic review and meta-analysis
Source: PLoS Med. 2020 Nov 12;17(11):e1003434. doi: 10.1371/journal.pmed.1003434 (PMC7660583; doi:10.1371/journal.pmed.1003434)
Supplement: S4 Appendix — (PDF) [file pmed.1003434.s004.pdf]

S4 Appendix: Main domains of the EPOC taxonomy of health systems

Category	Definition
Delivery arrangements	Changes in how, when and where healthcare is organized and delivered, and who delivers healthcare
Financial arrangements	Changes in how funds are collected, insurance schemes, how services are purchased, and the use of targeted financial incentives or disincentives
Governance arrangements	Rules or processes that affect the way in which powers are exercised, particularly with regard to authority, accountability, openness, participation, and coherence
Implementation strategies	Interventions designed to bring about changes in healthcare organizations, the behaviour of healthcare professionals or the use of health services by healthcare recipients

Source: Effective Practice and Organisation of Care (EPOC). EPOC taxonomy. 2015. https://epoc.cochrane.org/sites/epoc.cochrane.org/files/public/uploads/taxonomy/epoc_taxonomy.pdf.
