# Supplementary material for: Health system interventions for adults with type 2 diabetes in low- and middle-income countries: A systematic review and meta-analysis
Source: PLoS Med. 2020 Nov 12;17(11):e1003434. doi: 10.1371/journal.pmed.1003434 (PMC7660583; doi:10.1371/journal.pmed.1003434)
Supplement: S5 Appendix — (PDF) [file pmed.1003434.s005.pdf]

S5 Appendix: Characteristics of included studies

Study	Other reports	EPOC domain	Intervention type	Country	Setting	Intervention description	Duration (months)	Follow-up (months	Health professional(s)	Mortality	Quality of life	Cost-effectiveness	Glycemic change
Adibe[1]	Adibe 2013,[2] Adibe 2013[3]	Delivery arrangements	Pharmacist task sharing	Nigeria	Urban tertiary care hospital clinics	Pharmaceutical care program implemented by pharmacists and consisting of diabetes education (4 sessions of 90-120 minutes), assessing drug-related problems, and developing medication treatment plans. Glucometer and strips were provided to participants.	12	12	pharmacists	NR	Higher HRQOL as assessed by HUI23S4EN.40Q instrument (0.22 [95% CI, 0.19 to 0.25], p<0.001)	Incremental cost-utility ratio between groups of $571 per QALY gained favoring intervention	Lower mean HbA1c at 12 months (7.23% vs. 7.77%, p<0.001) in intervention compared to control arm, and no significant differences at baseline (p=0.318)
Akturan[4]	No	Implementation strategies	Physician clinical training alone	Turkey	Urban family medicine clinics	Physician training on therapeutic interviewing technique, delivered to patients 3 times at 3-month intervals. Details on physician trainers not provided.	6	6	physicians	NR	NR	NR	No significant within-group change in HbA1c in either arm
Ali[5]	Shah 2012,[6] Singh 2018,[7] Shah 2019[8]	Delivery arrangements, Implementation strategies	Multicomponent clinic-based intervention	India and Pakistan	Urban outpatient diabetes specialist clinics that were a mix of public, semi-private, and private facilities	Multicomponent quality improvement intervention. Non-physician care coordinators monitored laboratory and clinical follow-up (minimum every 3 months) and provided self-management education (at least once per month). Decision-support integrated into electronic health records based on clinical guidelines. Participants were responsible for treatment and follow-up costs.	28	28	care coordinators and physicians (endocrinologists)	12 deaths in intervention arm and 14 deaths in control arm (p-value not reported)	Higher quality of life in intervention group as assessed by change in EuroQol-5D score of 1.8 (95% CI, -0.2 to 3.9; p<0.001)	Incorporating patient-reported direct medical and non-medical costs, ICER for multiple risk factor control of $678 and 1% point reduction in HbA1c of $1,850. CEA from society perspective in progress.	Between-group reduction in mean HbA1c (-0.50% [95% CI, -0.69% to -0.32%]; p<0.001); within-group changes of -1.6% (95% CI, -1.8 to -1.4) in intervention and -1.2% (95% CI, -1.4 to -0.98) in control
Anzaldo-Campos[9]	Gilmer 2019[10]	Delivery arrangements, Implementation strategies	Multicomponent clinic-based intervention	Mexico	Urban public primary care unit	Intervention 1 (Project Dulce): Multidisciplinary care management led by trained clinicians and nurses, clinician guideline-based education consisting of 16 hours of training plus	10	10	peers, physicians, nurses	0 deaths in both arms	No significant between- or within-group differences as assessed by Diabetes 39 questionnaire	Compared to control, ICER for intervention 1 was $1635 and intervention 2 was $2220. ICER for intervention 2 was $4299	Within-group differences of -2.6% in intervention 1, -3.0% in intervention 2, and -1.3% in control; HbA1c changes were

Study	Other reports	EPOC domain	Intervention type	Country	Setting	Intervention description	Duration (months)	Follow -up (month s	Health professional(s)	Mortality	Quality of life	Cost-effectiveness	Glycemic change
						monthly case discussions, and peer diabetes education delivered in 8 weekly sessions during the first 2 months of the intervention. Intervention 2 (Project Dulce technology-enhanced with mobile tools): Intervention 1 plus remote glucose monitoring and cell phone education.						compared to intervention 1.	significant in intervention 1 (p=0.009) and 2 (p=0.001) compared to control, but no difference between intervention 1 and 2 (p=0.86)
Ayadurai[11]	No	Delivery arrangements	Pharmacist task sharing	Malaysia	Public primary care clinics	Multifaceted diabetes tool implemented by pharmacists using structured treatment goals, face-to-face visits, recommendations to prescribers, and telephone reminders	6	6	pharmacists	1 death in control arm, 0 deaths in intervention arm (p-value not reported)	NR	NR	Change in mean HbA1c in intervention of -1.59% (95% CI, -2.2 to -0.9) and control of -0.25 % (95% CI, -0.62 to 0.11), p<0.001 for difference in change
Barcelo[12]	No	Delivery arrangements, Implementation strategies	Multicomponent clinic-based intervention	Mexico	Urban health centers	Multidisciplinary teams collaborated on a quality improvement initiative based on the chronic care model and using PDSA cycles. Elements included education (patient self-management education and primary care provider education in foot care and diabetes management), peer support groups, specialist outreach, and case management.	18	18	multidisciplinary team members (physicians, nurses, nutritionists, psychologists) and peers	NR	NR	NR	Decreased within-group mean HbA1c in intervention (8.4% to 7.9%, p<0.01) but not control (8.7% to 8.6%, p=0.8)
Chao[13]	No	Delivery arrangements, Implementation strategies	Multicomponent clinic-based intervention	China	Urban setting	Integrated health management model, consisting of health record establishment, health evaluation (using software), and health management. The health management was implemented by community heath center	18	18	community heath center staff and managers	0 deaths in each arm	NR	NR	At 18 months, decreased within-group FBG in intervention group vs. control (-0.82 ± 1.9 mmol/l vs. 0.06 ± 2.36

Study	Other reports	EPOC domain	Intervention type	Country	Setting	Intervention description	Duration (months)	Follow-up (months	Health professional(s)	Mortality	Quality of life	Cost-effectiveness	Glycemic change
						staff, managers, and researchers. It included the following components: diet and psychological consultations, individually tailored exercise programs, education and skills training, follow-up visits, blood glucose monitoring, diabetes drug monitoring, and distribution of health promoting materials. Components of the intervention were administered at least once per month.							mmol/l, p=0.042).
Chapman[14]	Browning 2011,[15] Browning 2016[16], Chapman [17], Liu[18], Liu[19]	Delivery arrangements	Diabetes education or support alone	China	Urban government-operated community health station	Health coaching delivered by experienced clinicians (community doctors, nurses, and psychologists) via telephone (4-5 total calls) and in face-to-face encounters (6 total visits). Health coaching was based on motivational interviewing and incorporated local self management guidelines and recommendations. Participants were asked to identify the most productive place to start the discussion, which was then guided by health coaches. Medical fees waived.	18	18	physicians, nurses, and psychologists	NR	Similar changes quality of life changes as assessed by WHOQoL-BREF	NR	Similar change in mean HbA1c (-0.07% [95% CI, -0.53% to 0.39%]; p=0.769); within-group differences of −3.65% (95% CI, -3.92 to -3.37) in intervention and -3.38% (95% CI -3.67 to -3.08) in control
Chung[20]	No	Delivery arrangements	Pharmacist task sharing	Malaysia	Urban tertiary care hospital diabetes clinic	Pharmacist intervention consisting of medication review, self-management education, use of pill box and glucometer, and monthly telephone calls.	12	12	Pharmacists	NR	NR	NR	Between-group adjusted difference in HbA1c change of -0.486% (95% CI -0.177 to -0.795, p=0.002) favoring

Study	Other reports	EPOC domain	Intervention type	Country	Setting	Intervention description	Duration (months)	Follow -up (month s	Health professional(s)	Mortality	Quality of life	Cost-effectiveness	Glycemic change
													intervention arm.
DePue[21]	Hamid 2014,[22] Rao 2015,[23] Huang 2019[24]	Delivery arrangements	Case management by nurses	American Samoa	Government-funded community health center	Nurse and community health worker intervention focusing on case management, feedback to physicians, diabetes education, family support, and problem solving. Individual or family home visits were primarily utilized. Intervention contact intensity was weekly for high-risk, monthly for moderate-risk, and every 3 months for low-risk participants. Risk determined by HbA1c, BP, smoking status, alcohol use, and Patient Health Questionnaire (PHQ-9) depression scores at baseline	12	12	nurses and community health workers	2 deaths occurred in each arm (p-value between groups not reported)	NR	Compared to control, ICER for intervention was $1,121 per 1% HbA1c reduced and $13,191 per QALY gained	Between-group reduction in adjusted mean HbA1c (-0.53% [SE 0.21, p=0.03); unadjusted 12-month HbA1c of 9.6% to 9.3% in intervention and 10.0% to 10.0% in control. Post-trial data suggested that HbA1c plateaued or increased in years following intervention.
Fairall[25]	No	Delivery arrangements, Governance arrangements, Implementation strategies	Nurse task sharing	South Africa	60% urban, 20% peri-urban, and 20% rural outpatient primary clinics in public sector	Implementation and training (8 sessions over 4 months) of algorithmic clinical management tool tailored to nurses, expanding prescribing provisions (including glibenclamide and gliclazide for diabetes), and quarterly follow-up workshops for nurses.	14	14	Nurses	Similar mortality in each group (including among diabetes participants of whom 31 died in control arm and 30 in intervention arm)	Similar quality of life as assessed by EuroQol 5D (including diabetes and non-diabetes participants)	NR	Similar between-group change in mean HbA1c (0.21% [95% CI, -0.43% to 0.85%]; p=0.508); within-group differences of 0.0% in intervention and -0.2% in control
Gillani[26]	No	Delivery arrangements	Pharmacist task sharing	Malaysia	Urban, public-sector diabetes clinics and hospital	Pharmacist intervention including self-management education, development of glucose-monitoring schedule, and weekly home visits.	6	6	Pharmacists	NR	NR	NR	Mean HbA1c within-group differences in intervention 1 of -0.9%, intervention 2 of -2.7%, and control of 0.7%. Greater HbA1c improvement in pharmacist

Study	Other reports	EPOC domain	Intervention type	Country	Setting	Intervention description	Duration (months)	Follow -up (month s	Health professional(s)	Mortality	Quality of life	Cost-effectiveness	Glycemic change
													intervention arm than telemonitoring and control arms (p<0.001). Statistical significance for other comparisons not reported.
Goruntla[27]	No	Delivery arrangements	Pharmacist task sharing	India	Outpatient medical department of secondary referral hospital	Pharmacist intervention consisting of medication review, self-management education (delivered in 3 counseling sessions), and daily text message reminders for medication intake and aerobic exercise.	6	6	Pharmacists	NR	NR	NR	Lower mean HbA1c at 6 months in intervention vs. control (6.91% vs. 7.49%, p=0.002); no difference observed at baseline (7.79% vs. 7.78%, p=0.97)
Guo[28]	No	Delivery arrangements	Diabetes education or support alone	China	Community health center	Nurse-led team management intervention focused on diabetes education and support. The intervention consisted of 12 nurse-delivered telephone and home-based family visits, 6 health lectures, and 6 consultations with a diabetes expert over a period of 12 months.	12	12	nurses, physicians, nutritionists	5 deaths in intervention arm; 3 deaths in control arm	NR	NR	At 12 months, lower mean HbA1c in intervention vs control (7.18% ± 1.00 vs 7.79% ± 1.33, p=0.002); no difference observed at baseline (7.76% ± 1.71 vs 7.64% ± 1.50, p=0.698).
Jaipakdee[29]	No	Delivery arrangements	Diabetes education or support alone	Thailand	Urban public health centers	Training health center nurses and implementation of diabetes self-management support using computer-assisted instruction. Training consisted of a two-day intensive course, and nurses implemented the material in 6 monthly sessions of 3 hours each. The material included diabetes education, skill-learning, and	6	6	nurses	NR	Between-group increase in adjusted mean Diabetes QOL Brief Clinical Inventory score (1.41 [95% CI, 0.69 to 2.12], p<0.001)	NR	Between-group reduction in adjusted mean HbA1c (-0.14% [95% CI, -0.26% to -0.02%, p=0.025); unadjusted within-group change in HbA1c in intervention (8.2% to 7.8%, p not reported)

Study	Other reports	EPOC domain	Intervention type	Country	Setting	Intervention description	Duration (months)	Follow -up (month s	Health professional(s)	Mortality	Quality of life	Cost-effectiveness	Glycemic change
						psychological support intended to help patients manage their conditions and make lifestyle changes.							and control (8.5% to 8.2%, p not reported)
Jarab[30]	No	Delivery arrangements	Pharmacist task sharing	Jordan	Urban tertiary care hospital diabetes clinic	Clinical pharmacist intervention consisting of medication recommendations and review, self-management education, and 8 weekly telephone calls (discussing prescribed therapy, adherence to treatment plan, and patient concerns).	6	6	pharmacists	NR	NR	NR	Change in mean HbA1c in intervention of -0.8% (95% CI, -1.6 to 0.1) and control of 0.1% (95% CI, -0.4 to 0.7), p=0.019 for difference in change
Javaid[31]	No	Delivery arrangements	Pharmacist task sharing	Pakistan	Urban primary care clinic	Pharmacist intervention consisting of medication review, patient education and counseling, discussion of medication adherence and treatment goals, lifestyle recommendations, instructions for self-monitoring of blood glucose, assessment of diabetic complications and foot care, and counseling for personal care and hygiene. Patients met with pharmacists every 4 weeks and additionally reviewed individualized treatment recommendations with physicians at routine clinical visits every 3 months.	9	9	pharmacists	0 deaths in intervention arm; 1 deaths in control arm	NR	NR	Within-group reduction in mean HbA1c from baseline to 9 months for both intervention (11.0% ± 1.7 to 7.7% ± 0.9 , p<0.001) and control (10.7% ± 1.7 to 9.7% ± 1.3, p<0.001). Lower mean HbA1c at 9 months for intervention vs control (p<0.001).
Khan[32]	Khan 2018[33]	Delivery arrangements, Implementation strategies	Multicomponent clinic-based intervention	Pakistan	Public rural health centers (9) and sub-district hospitals (5)	Integrated diabetes care package with comparator components plus additional case management including standardized clinical treatment guide, lifestyle education materials, and monthly mobile phone	9	9	physicians and allied staff	0 deaths in both arms	NR	NR	Similar adjusted change in mean HbA1c (-0.57% [95% CI, -1.44 to 0.29]; p=0.17); within-group differences of −2.26% (95%

Study	Other reports	EPOC domain	Intervention type	Country	Setting	Intervention description	Duration (months)	Follow -up (month s	Health professional(s)	Mortality	Quality of life	Cost-effectiveness	Glycemic change
						appointment review and reminders. Drugs were available to participants in both arms, though with some top up in intervention facilities.							CI, -2.99 to -1.53) in intervention and -1.44% (95% CI -2.34 to -0.54) in control
Khetan[34]	Khetan 2017,[35] Khetan 2018[36]	Delivery arrangements	Diabetes education or support alone	India	Home visits	For patients with diabetes, community health workers conducted in-home sessions focused on diabetes education and counseling, lifestyle and behavior changes (including the promotion of health care-seeking behavior), and medication adherence. Visits were conducted approximately every 2 months over a period of 18 months. Community health workers were trained in diabetes counseling over 1 to 2 weeks, for 3 hours per day.	24	18	community health workers	NR	NR	NR	No between-group adjusted difference in FBG in intervention compared to control (-21.3 mg/dl [95% CI -61.0 to 18.3], p=0.29)
Kim[37]	No	Delivery arrangements	Internet-based glucose telemonitoring	China	Urban hospital diabetes clinic	Implementation of an internet-based glucose management system in which participants uploaded glucose data at least twice per week and received feedback from nurses regarding glucose control. Nurses provided recommendations weekly for the first 3 months, and biweekly for the remaining 3 months.	6	6	nurses	0 deaths in each arm	NR	NR	Lower mean HbA1c at 6 months in intervention vs. control (6.7 vs. 7.4, p<0.01); no difference observed at baseline (7.9 vs. 8.0, p>0.05)
Kong[38]	No	Delivery arrangements, Implementation strategies	Multicomponent clinic-based intervention	China	Community health centers	Chronic care model-based intervention including health system stimulation, self-management support, decision support including implementation of clinical guidelines,	9	9	physicians, health managers, and public health assistants	NR	Between-group differences in adjusted increase in SF-36 physical health summary (mean 3.31; 95% CI, 1.22	NR	No between-group adjusted difference in mean HbA1c (-0.21% [95% CI, -0.77 to 0.38]; p=0.08); within-group decrease in

Study	Other reports	EPOC domain	Intervention type	Country	Setting	Intervention description	Duration (months)	Follow -up (months	Health professional(s)	Mortality	Quality of life	Cost-effectiveness	Glycemic change
						delivery system design, and clinical information system. The chronic care model specifies a team-centered approach to care, and each team included a physician, health manager, and a public health assistant.					to 5.39); no difference in mental health summary		HbA1c in intervention (7.17% to 6.60%, p<0.001) but not control (7.91% to 7.45%, p=0.28)
Lee[39]	Lee 2016,[40] Lee 2017[41]	Delivery arrangements	Internet-based glucose telemonitoring	Malaysia	Government primary care clinics	Glucose telemonitoring intervention mainly consisting of upload from gluco-telemeter given to participants to online portal (at least 6 times per week) with automated feedback. Physicians were notified if 3 consecutive readings of hypoglycemia or hyperglycemia were recorded, and may recommend treatment or medication changes. Participants also received monthly communications from the research team on self-management skills, blood glucose control, and the importance of medication adherence aimed at educating and motivating patients	6	12	physicians	0 deaths in each arm	No between-group difference in quality of life as assessed by EuroQol-5D questionnaire	NR	At 52 weeks, no between-group difference in HbA1c reduction in intervention vs. control (-0.03% [95% CI -0.07 to 0.02], p=0.226). Within-group HbA1c reductions in intervention and control were -0.33% [95% CI -0.37 to -0.29] and -0.30% [95% CI -0.33 to -0.27], respectively.
Mash[42]	Mash 2012,[43] Mash 2015[44]	Delivery arrangements	Diabetes education or support alone	South Africa	Urban public sector community health centers	Training and implementation of group diabetes education consisting of 4 sessions of 60 minutes duration, focused on understanding diabetes, living a healthy lifestyle, understanding the medication, and avoiding complications. Sessions were delivered by health promoters, lay people employed by community health centers trained in motivational	4	12	community health workers	NR	Higher quality of life in intervention group as assessed by change in EuroQol-5D score of 1.8 (95% CI, -0.2 to 3.9; p<0.001)	Based on improvements in blood pressure, incremental cost-effectiveness ratio estimated at US$1862 per QALY	No adjusted between-group difference in mean HbA1c (0.01% [95% CI, -0.27 to 0.28]; p=0.967); unadjusted within-group decrease in HbA1c in intervention (8.9% to 8.4%, p not reported) and control

Study	Other reports	EPOC domain	Intervention type	Country	Setting	Intervention description	Duration (months)	Follow -up (month s	Health professional(s)	Mortality	Quality of life	Cost-effectiveness	Glycemic change
						interviewing techniques. Groups were between 10-15 people.							(9.3% to 8.8%, p not reported)
Mourao[45]	No	Delivery arrangements	Pharmacist task sharing	Brazil	Primary care units in public sector	Pharmacist-delivered self-management education (6 monthly appointments) and suggestions to physicians for pharmacotherapy changes.	6	6	pharmacists	NR	NR	NR	Greater within-group reduction in mean HbA1c at 6 months in intervention vs. control (-0.6% vs. 0.7%, p=0.001)
Neto[46]	No	Delivery arrangements	Pharmacist task sharing	Brazil	Public primary health care units	Pharmaceutical care intervention consisting of individual follow-up and group educative activities. The individual follow-ups were implemented by 4 pharmacists at a frequency of 1 visit every 6 months, and included assessment of non-compliance problems, discussions with patients and family about the role of medication in their health status, suggestions to physicians concerning new drug regimens, and the preparation visual reminders for when medication was taken. Pharmacists also worked with nurses and general practitioners to establish plans and goals for drug therapy.	36	36	pharmacists	NR	NR	NR	At 36 months, greater within-group reduction in HbA1c in intervention (-0.7% [95% CI -0.9 to 0.6], p<0.001) compared to control (0.0% [95% CI -0.1 to 0.1], p=0.885), with between-group p<0.001.
Paz-Pacheco[47]	No	Delivery arrangements	Diabetes education or support alone	Philippines	Rural agricultural town	Training and implementation of a group peer educator-led diabetes self-management education. Peers were community members trained in a 2-day workshop. An 8-module curriculum was delivered in village health centers with 6-15 participants per session.	1	6	peers	2 deaths in intervention arm and 1 death in control arm (p-value not reported)	NR	NR	Greater within-group reduction in median HbA1c at 6 months in intervention vs. control (-0.5% vs. 0.25%, p=0.019); lower median HbA1c at 6 months

Study	Other reports	EPOC domain	Intervention type	Country	Setting	Intervention description	Duration (months)	Follow -up (month s	Health professional(s)	Mortality	Quality of life	Cost-effectiveness	Glycemic change
						2 modules were taught per session. Sessions were delivered weekly and lasted approximately 1 hour.							(6.45% vs. 7.6%, p=0.01)
Phumipamorn [48]	No	Delivery arrangements	Pharmacist task sharing	Thailand	Community hospital	Pharmacist intervention comprising 4 visits with patients at 2-month intervals, which consisted of prescription refills, discussion of medication use and pill count, and diabetes education concerning lifestyle and diet.	8	8	pharmacists	NR	NR	NR	No between-group difference (p=0.56) in mean HbA1c reduction in intervention (-0.8% [95% CI -1.3 to 0.4], p=0.001) compared to control (-0.6% [95% CI -1.0 to -0.19], p=0.006).
Prabhakaran[49]	Jha 2017[50]	Delivery arrangements, Implementation strategies	Multicomponent clinic-based intervention	India	Rural referral health centers	Mobile health clinic-based system including electronic health record storage, decision support, and short service reminders to patients and clinical team. Nurses and physicians were trained in the use of the mobile health system and also received training on clinical management guidelines for hypertension and diabetes.	12	12	nurses and physicians	21 deaths in control arm and 34 deaths in intervention arm (p-value not reported)	NR	NR	Similar change in adjusted mean HbA1c (0.08% [95% CI, -0.27% to 0.44]; p=0.660); within-group differences of -0.58% in control and -0.48% in intervention (CI not reported)
Ramli[51]	Ramli 2014[52]	Delivery arrangements, Implementation strategies	Multicomponent clinic-based intervention	Malaysia	Urban and suburban public primary care clinics	Multicomponent intervention based on the Chronic Care Model and consisting of obligatory (multidisciplinary chronic disease management teams led by family medicine specialists, support of self-management, utilization of clinical practice guidelines) and optional components (clinical information	12	12	multidisciplinary teams led by family medicine specialists	6 deaths in intervention arm and 3 deaths in control arm (p-value not reported)	NR	NR	Greater within-group reduction in mean HbA1c at 6 months in intervention vs. control (-0.1% vs. 0.2%, p=0.003 of intervention term in model)

Study	Other reports	EPOC domain	Intervention type	Country	Setting	Intervention description	Duration (months)	Follow -up (months	Health professional(s)	Mortality	Quality of life	Cost-effectiveness	Glycemic change
						system and conducting clinical audits, utilizing community resources).							
Reutens[53]	No	Implementation strategies	Physician clinical training alone	China, Hong Kong, South Korea, Vietnam, Philippines, Indonesia, Singapore, Taiwan, Thailand, Malaysia	Urban primary care practices in both private and public sector	GP education (2 sessions held by the national investigator) and reminders about clinical guidelines and treatment targets. Patients received written materials on metabolic targets.	12	12	physicians	1 death reported in control arm and none in intervention arm	NR	NR	At 12 months in those with HbA1c ≥6.5, no between-group difference in mean HbA1c change in intervention (-0.17% [95% CI, -0.33 to -0.01]) compared with control (-0.28% [95% CI, -0.45 to -0.11]), p=0.332
Saleh[54]	No	Delivery arrangements, Implementation strategies	mHealth screening and quality improvement	Lebanon	Rural (10) and refugee (6) primary health care centers	Multicomponent mobile health intervention including NCD screening and referral using mass messaging, at least weekly targeted SMS educational messages developed by a family physician, targeted appointment reminders, and online tools for physicians and nurses focusing on treatment guidelines, communication strategies, and knowledge sharing.	12	12	physicians and nurses	NR	NR	NR	Within-group decrease in mean HbA1c (-0.87, p<0.01) in intervention arm but not control arm (-0.22, p=0.41)
Sarayani[55]	No	Delivery arrangements	Pharmacist task sharing	Iran	Urban referral pharmacy	Pharmacist intervention consisting of 16 telephone calls over 3 months. Topics covered included self-management, blood glucose trends, and drug therapy problems.	3	9	pharmacists	NR	NR	NR	No between-group difference in mean in HbA1c in intervention compared with control (p=0.78); within-group change in intervention of 7.84% to 6.96% (p not reported) and control of

Study	Other reports	EPOC domain	Intervention type	Country	Setting	Intervention description	Duration (months)	Follow -up (month s	Health professional(s)	Mortality	Quality of life	Cost-effectiveness	Glycemic change
													8.16% to 7.26% (p not reported)
Shen[56]	No	Delivery arrangements	Pharmacist task sharing	China	"Community Health Service Centres (CHSC)"	Pharmaceutical care intervention including (1) personalized medication cards, (2) home visits, (3) availability for phone calls and text messages, (4) pharmacy window service, (5) health education, and (6) establishment of pharmaceutical record. The pharmacists implementing the interventions were senior pharmacy professionals and technical personnel.	12	12	pharmacists	0 deaths in intervention arm and 2 deaths in control arm	NR	NR	FBG: At 12 months, decrease in intervention arm (-0.65±2.31 mmol/L, p=0.019) but not control arm (-0.21±1.96 mmol/L, p=0.356); no baseline difference between groups. HbA1c: At 12 months, decrease in intervention arm (-0.42±1.27%, p=0.006) but not control arm (-0.17±1.04%, p=0.159); no baseline difference between groups.
Sriram[57]	No	Delivery arrangements	Pharmacist task sharing	India	Private tertiary care hospital	Pharmaceutical care which included medication counseling, instructions on dietary regulation, exercise, and other lifestyle modifications	8	8	pharmacists	0 deaths in each arm	As assessed by ADDQOL questionnaire, the intervention group improved from baseline to end (-2.156 to 1.41, p<0.01) while the control group showed no improvement (-1.899 to -1.974, p=NS)	NR	Within-group reduction in mean HbA1c from baseline to 8 months in intervention arm (8.44% to 6.73%, p<0.01) but not control (9.03% to 8.31%, p>0.05).
Tutino[58]	No	Delivery arrangements	Case management by nurses	China	Tertiary hospitals	Nurse-coordinated follow-up including documentation of modifiable risk factors,	12	13	nurses	6 deaths occurred in each arm (p-value	No significant between-group difference as	NR	Similar mean change in HbA1c in each group

Study	Other reports	EPOC domain	Intervention type	Country	Setting	Intervention description	Duration (months)	Follow -up (month s	Health professional(s)	Mortality	Quality of life	Cost-effectiveness	Glycemic change
						hypoglycemia, and key events, in addition to web-based portal for comprehensive clinical assessment of a patient and including templates for periodic assessment, risk stratification, personalized reporting, and automated decision support (Joint Asia Diabetes Evaluation [JADE])				between groups not reported)	assessed by EQ-5D index		(DIAMOND -0.69% vs. JADE -0.62%, p=0.473)
Van Olmen[59]	Van Olmen 2013,[60] Van Olmen 2015,[61] Van Olmen 2017[62]	Delivery arrangements	Diabetes education or support alone	Democratic Republic of Congo, Cambodia, and the Philippines	Faith-based primary care facilities (DRC), NGO-supported community-based peer educator network (Cambodia), community diabetes health worker program (Philippines)	Mobile phone DSMS intervention consisting of free mobile phone for participants and educators, receipt of self-management and support SMS messages at least 2 times per week, and educator budget for increased calls/SMS communication with participants. Educators were specialized nurses (Congo); community-based peers (Cambodia); and trained CHWs, nursing aides, or midwives (Philippines).	24	24	nurses, peers, and community health workers	30 deaths in intervention arm and 36 deaths in control arm	NR	NR	Similar between-group adjusted change in mean HbA1c (-0.06 with SE of 0.11). No significant difference in proportion of patients with controlled diabetes (HbA1c < 7.0%) at 2 years between intervention and control (33.9% vs 31.2%, p = 0.39).
Wang[63]	Xu 2014[64], Liu[65]	Delivery arrangements	Diabetes education or support alone	China	Community health clinics	Health literacy intervention consisting of a toolkit with 24 educational modules designed to improve diabetes self-management in areas that include diet, exercise, foot care, glucose monitoring, medication management, and enhanced diabetes logging. At each clinic visit (typically once or twice per month), physicians spent approximately 5-7 minutes to share at least	12	24	physicians, nurses, and health educators	NR	NR	NR	At 24 months, adjusted within-group reduction in mean HbA1c in intervention arm (-0.68%, p<0.001) but not control (0.06%, p=0.66)

Study	Other reports	EPOC domain	Intervention type	Country	Setting	Intervention description	Duration (months)	Follow -up (month s	Health professional(s)	Mortality	Quality of life	Cost-effectiveness	Glycemic change
						two components from the toolkit with the patient, and nurses or health educators phoned the patients afterward. Prior to starting the intervention, physicians, nurses, and health educators were also trained in use of the Toolkit and heath communication skills.							
Wishah[66]	No	Delivery arrangements	Pharmacist task sharing	Jordan	Urban tertiary care hospital diabetes clinic	Clinical pharmacist intervention consisting of medication review and recommendation to physician, self-management education, and telephone calls	6	6	pharmacists	NR	NR	NR	Lower mean HbA1c in intervention vs. control (7.2 vs. 7.9, p=0.01); mean HbA1c was higher in intervention at baseline (8.9 vs. 8.2, p=0.01)
Zhong[67]	No	Delivery arrangements	Diabetes education or support alone	China	Primary care community health services centers	Training peer leaders and implementation of a group peer support system. Peers received 3 days training and led groups of 10-15 participants for 12 biweekly education meetings and 12 biweekly discussion meetings over 6 months. Peer leaders also led or encouraged informal activities (e.g., walking and tai chi groups) among group members, promoted care through community health service centers, and provided informal individual support.	6	10	peers	NR	NR	NR	Greater improvement in FBG glucose (mg/dL) in intervention vs. control (138 to 121 vs. 115 to 120, p<0.001)

References list of studies including "Other reports":

1. Adibe MO, Obinna UP, Uchenna IN, Michael UC, Aguwa CN. Effects of an additional pharmaceutical care intervention versus usual care on clinical outcomes of Type 2 diabetes patients in Nigeria: A comparative study. Scientific Research and Essays. 2014; 9(12):548-56.

2. Adibe MO, Aguwa CN, Ukwe CV. Cost-Utility Analysis of Pharmaceutical Care Intervention Versus Usual Care in Management of Nigerian Patients with Type 2 Diabetes. Value Health Reg Issues. 2013; 2(2):189-98. http://dx.doi.org/10.1016/j.vhri.2013.06.009 PMID: 29702864

3. Adibe MO, Ukwe CV, Aguwa CN. The Impact of Pharmaceutical Care Intervention on the Quality of Life of Nigerian Patients Receiving Treatment for Type 2 Diabetes. Value Health Reg Issues. 2013; 2(2):240-7. http://dx.doi.org/10.1016/j.vhri.2013.06.007 PMID: 29702871

4. Akturan S, Kaya CA, Unalan PC, Akman M. The effect of the BATHE interview technique on the empowerment of diabetic patients in primary care: A cluster randomised controlled study. Prim Care Diabetes. 2017; 11(2):154-61. http://dx.doi.org/10.1016/j.pcd.2016.12.003 PMID: 28122690

5. Ali MK, Singh K, Kondal D, Devarajan R, Patel SA, Shivashankar R, et al. Effectiveness of a Multicomponent Quality Improvement Strategy to Improve Achievement of Diabetes Care Goals: A Randomized, Controlled Trial. Ann Intern Med. 2016; 165(6):399-408. http://dx.doi.org/10.7326/M15-2807 PMID: 27398874

6. Shah S, Singh K, Ali MK, Mohan V, Kadir MM, Unnikrishnan AG, et al. Improving diabetes care: multi-component cardiovascular disease risk reduction strategies for people with diabetes in South Asia--the CARRS multi-center translation trial. Diabetes Res Clin Pract. 2012; 98(2):285-94. http://dx.doi.org/10.1016/j.diabres.2012.09.023 PMID: 23084280

7. Singh K, Johnson L, Devarajan R, Shivashankar R, Sharma P, Kondal D, et al. Acceptability of a decision-support electronic health record system and its impact on diabetes care goals in South Asia: a mixed-methods evaluation of the CARRS trial. Diabet Med. 2018; 35(12):1644-54. http://dx.doi.org/10.1111/dme.13804 PMID: 30142228

8. Shah MK, Kondal D, Patel SA, Singh K, Devarajan R, Shivashankar R, et al. Effect of a multicomponent intervention on achievement and improvements in quality-of-care indices among people with Type 2 diabetes in South Asia: the CARRS trial. Diabet Med. 2019. http://dx.doi.org/10.1111/dme.14124 PMID: 31479537

9. Anzaldo-Campos MC, Contreras S, Vargas-Ojeda A, Menchaca-Diaz R, Fortmann A, Philis-Tsimikas A. Dulce Wireless Tijuana: A Randomized Control Trial Evaluating the Impact of Project Dulce and Short-Term Mobile Technology on Glycemic Control in a Family Medicine Clinic in Northern Mexico. Diabetes Technol Ther. 2016; 18(4):240-51. http://dx.doi.org/10.1089/dia.2015.0283 PMID: 26914371

10. Gilmer T, Burgos JL, Anzaldo-Campos MC, Vargas-Ojeda A. Cost-Effectiveness of a Technology-Enhanced Diabetes Care Management Program in Mexico. Value Health Reg Issues. 2019; 20:41-6. http://dx.doi.org/10.1016/j.vhri.2018.12.006 PMID: 30856542

11. Ayadurai S, Sunderland VB, Tee LBG, Md Said SN, Hattingh HL. Structured tool to improve clinical outcomes of type 2 diabetes mellitus patients: A randomized controlled trial. J Diabetes. 2018; 10(12):965-76. http://dx.doi.org/10.1111/1753-0407.12799 PMID: 29877609

12. Barcelo A, Cafiero E, de Boer M, Mesa AE, Lopez MG, Jimenez RA, et al. Using collaborative learning to improve diabetes care and outcomes: the VIDA project. Prim Care Diabetes. 2010; 4(3):145-53. http://dx.doi.org/10.1016/j.pcd.2010.04.005 PMID: 20478753

13. Chao J, Yang L, Xu H, Yu Q, Jiang L, Zong M. The effect of integrated health management model on the health of older adults with diabetes in a randomized controlled trial. Arch Gerontol Geriatr. 2015; 60(1):82-8. http://dx.doi.org/10.1016/j.archger.2014.10.006 PMID: 25456892

14. Chapman A, Browning CJ, Enticott JC, Yang H, Liu S, Zhang T, et al. Effect of a Health Coach Intervention for the Management of Individuals With Type 2 Diabetes Mellitus in China: A Pragmatic Cluster Randomized Controlled Trial. Front Public Health. 2018; 6:252. http://dx.doi.org/10.3389/fpubh.2018.00252 PMID: 30283767

15. Browning C, Chapman A, Cowlishaw S, Li Z, Thomas SA, Yang H, et al. The Happy Life Club study protocol: a cluster randomised controlled trial of a type 2 diabetes health coach intervention. BMC Public Health. 2011; 11:90. http://dx.doi.org/10.1186/1471-2458-11-90 PMID: 21303564

16. Browning C, Chapman A, Yang H, Liu S, Zhang T, Enticott JC, et al. Management of type 2 diabetes in China: the Happy Life Club, a pragmatic cluster randomised controlled trial using health coaches. BMJ Open. 2016; 6(3):e009319. http://dx.doi.org/10.1136/bmjopen-2015-009319 PMID: 26944692

17. Chapman A. An Examination of the Effectiveness of Psychological Interventions for the Management of Type 2 Diabetes Mellitus in China: Monash University; 2017.

18. Liu S, Yang H, Browning C. Process analysis of motivational interviewing intervention by primary healthcare setting staffs in China — the Happy Life Club. Chinese General Practice. 2015; 18(19): 2241-8.

19. Liu S, Yang H, Browning C. The Influence of Family Responsibility on the Self-management Behavior of Patients With Type 2 Diabetes Mellitus—The Happy Life Club. Chinese General Practice. 2015; 18(13):1477-82.

20. Chung WW, Chua SS, Lai PS, Chan SP. Effects of a pharmaceutical care model on medication adherence and glycemic control of people with type 2 diabetes. Patient Prefer Adherence. 2014; 8:1185-94. http://dx.doi.org/10.2147/PPA.S66619 PMID: 25214772

21. DePue JD, Dunsiger S, Seiden AD, Blume J, Rosen RK, Goldstein MG, et al. Nurse-community health worker team improves diabetes care in American Samoa: results of a randomized controlled trial. Diabetes Care. 2013; 36(7):1947-53. http://dx.doi.org/10.2337/dc12-1969 PMID: 23393217

22. Hamid S, Dunsiger S, Seiden A, Nu'usolia O, Tuitele J, DePue JD, et al. Impact of a diabetes control and management intervention on health care utilization in American Samoa. Chronic Illn. 2014; 10(2):122-34. http://dx.doi.org/10.1177/1742395313502367 PMID: 24085749

23. Rao M, DePue JD, Dunsiger S, Elsayed M, Nu'usolia O, McGarvey ST. Long-Term Impact of a Community Health Worker Intervention on Diabetes Control in American Samoa. Prev Chronic Dis. 2015; 12:E180. http://dx.doi.org/10.5888/pcd12.150160 PMID: 26491815

24. Huang SJ, Galarraga O, Smith KA, Fuimaono S, McGarvey ST. Cost-effectiveness analysis of a cluster-randomized, culturally tailored, community health worker home-visiting diabetes intervention versus standard care in American Samoa. Hum Resour Health. 2019; 17(1):17. http://dx.doi.org/10.1186/s12960-019-0356-6 PMID: 30836964

25. Fairall LR, Folb N, Timmerman V, Lombard C, Steyn K, Bachmann MO, et al. Educational Outreach with an Integrated Clinical Tool for Nurse-Led Non-communicable Chronic Disease Management in Primary Care in South Africa: A Pragmatic Cluster Randomised Controlled Trial. PLoS Med. 2016; 13(11):e1002178. http://dx.doi.org/10.1371/journal.pmed.1002178 PMID: 27875542

26. Gillani SW. Determining Effective Diabetic Care; A Multicentre - Longitudinal Interventional Study. Curr Pharm Des. 2016; 22(42):6469-76. http://dx.doi.org/10.2174/1381612822666160813235704 PMID: 27526787

27. Goruntla N, Mallela V, Nayakanti D. Impact of Pharmacist-directed Counseling and Message Reminder Services on Medication Adherence and Clinical Outcomes in Type 2 Diabetes Mellitus. J Pharm Bioallied Sci. 2019; 11(1):69-76. http://dx.doi.org/10.4103/jpbs.JPBS_211_18 PMID: 30906142

28. Guo Z, Liu J, Zeng H, He G, Ren X, Guo J. Feasibility and efficacy of nurse-led team management intervention for improving the self-management of type 2 diabetes patients in a Chinese community: a randomized controlled trial. Patient Prefer Adherence. 2019; 13:1353-62. http://dx.doi.org/10.2147/PPA.S213645 PMID: 31616135

29. Jaipakdee J, Jiamjarasrangsi W, Lohsoonthorn V, Lertmaharit S. Effectiveness of a self-management support program for Thais with type 2 diabetes: Evaluation according to the RE-AIM framework. Nurs Health Sci. 2015; 17(3):362-9. http://dx.doi.org/10.1111/nhs.12198 PMID: 25818472

30. Jarab AS, Alqudah SG, Mukattash TL, Shattat G, Al-Qirim T. Randomized controlled trial of clinical pharmacy management of patients with type 2 diabetes in an outpatient diabetes clinic in Jordan. J Manag Care Pharm. 2012; 18(7):516-26. http://dx.doi.org/10.18553/jmcp.2012.18.7.516 PMID: 22971205

31. Javaid Z, Imtiaz U, Khalid I, Saeed H, Khan RQ, Islam M, et al. A randomized control trial of primary care-based management of type 2 diabetes by a pharmacist in Pakistan. BMC Health Serv Res. 2019; 19(1):409. http://dx.doi.org/10.1186/s12913-019-4274-z PMID: 31234840

32. Khan MA, Walley JD, Khan N, Hicks J, Ahmed M, Khan SE, et al. Effectiveness of an integrated diabetes care package at primary healthcare facilities: a cluster randomised trial in Pakistan. BJGP Open. 2018; 2(4):bjgpopen18X101618. http://dx.doi.org/10.3399/bjgpopen18X101618 PMID: 30723803

33. Khan MA, Walley JD, Ali S, King R, Khan SE, Khan N, et al. Process evaluation of integrated diabetes management at primary healthcare facilities in Pakistan: a mixed-methods study. BJGP Open. 2018; 2(4):bjgpopen18X101612. http://dx.doi.org/10.3399/bjgpopen18X101612 PMID: 30723798

34. Khetan A, Zullo M, Rani A, Gupta R, Purushothaman R, Bajaj NS, et al. Effect of a Community Health Worker-Based Approach to Integrated Cardiovascular Risk Factor Control in India: A Cluster Randomized Controlled Trial. Glob Heart. 2019; 14(4):355-65. http://dx.doi.org/10.1016/j.gheart.2019.08.003 PMID: 31523014

35. Khetan A, Purushothaman R, Zullo M, Gupta R, Hejjaji V, Agarwal S, et al. Rationale and design of a cluster-randomized controlled trial to evaluate the effects of a community health worker-based program for cardiovascular risk factor control in India. Am Heart J. 2017; 185:161-72. http://dx.doi.org/10.1016/j.ahj.2016.10.027 PMID: 28267470

36. Khetan A, Patel T, Hejjaji V, Barbhaya D, Mohan SKM, Josephson R, et al. Role development of community health workers for cardiovascular disease prevention in India. Eval Program Plann. 2018; 67:177-83. http://dx.doi.org/10.1016/j.evalprogplan.2018.01.006 PMID: 29407939

37. Kim HS, Sun C, Yang SJ, Sun L, Li F, Choi IY, et al. Randomized, Open-Label, Parallel Group Study to Evaluate the Effect of Internet-Based Glucose Management System on Subjects with Diabetes in China. Telemed J E Health. 2016; 22(8):666-74. http://dx.doi.org/10.1089/tmj.2015.0170 PMID: 26938489

38. Kong JX, Zhu L, Wang HM, Li Y, Guo AY, Gao C, et al. Effectiveness of the Chronic Care Model in Type 2 Diabetes Management in a Community Health Service Center in China: A Group Randomized Experimental Study. J Diabetes Res. 2019; 2019:6516581. http://dx.doi.org/10.1155/2019/6516581 PMID: 30719455

39. Lee JY, Chan CKY, Chua SS, Ng CJ, Paraidathathu T, Lee KKC, et al. Telemonitoring and Team-Based Management of Glycemic Control on People with Type 2 Diabetes: a Cluster-Randomized Controlled Trial. J Gen Intern Med. 2020; 35(1):87-94. http://dx.doi.org/10.1007/s11606-019-05316-9 PMID: 31512187

40. Lee JY, Chan CK, Chua SS, Ng CJ, Paraidathathu T, Lee KK, et al. Intervention for Diabetes with Education, Advancement and Support (IDEAS) study: protocol for a cluster

randomised controlled trial. BMC Health Serv Res. 2016; 16(1):524. http://dx.doi.org/10.1186/s12913-016-1782-y PMID: 27683021

41. Lee JY, Wong CP, Tan CSS, Nasir NH, Lee SWH. Telemonitoring in fasting individuals with Type 2 Diabetes Mellitus during Ramadan: A prospective, randomised controlled study. Sci Rep. 2017; 7(1):10119. http://dx.doi.org/10.1038/s41598-017-10564-y PMID: 28860546

42. Mash RJ, Rhode H, Zwarenstein M, Rollnick S, Lombard C, Steyn K, et al. Effectiveness of a group diabetes education programme in under-served communities in South Africa: a pragmatic cluster randomized controlled trial. Diabet Med. 2014; 31(8):987-93. http://dx.doi.org/10.1111/dme.12475 PMID: 24766179

43. Mash B, Levitt N, Steyn K, Zwarenstein M, Rollnick S. Effectiveness of a group diabetes education programme in underserved communities in South Africa: pragmatic cluster randomized control trial. BMC Family Practice. 2012; 13(1):126. http://dx.doi.org/10.1186/1471-2296-13-126

44. Mash R, Kroukamp R, Gaziano T, Levitt N. Cost-effectiveness of a diabetes group education program delivered by health promoters with a guiding style in underserved communities in Cape Town, South Africa. Patient Educ Couns. 2015; 98(5):622-6. http://dx.doi.org/10.1016/j.pec.2015.01.005 PMID: 25641665

45. Mourao AO, Ferreira WR, Martins MA, Reis AM, Carrillo MR, Guimaraes AG, et al. Pharmaceutical care program for type 2 diabetes patients in Brazil: a randomised controlled trial. Int J Clin Pharm. 2013; 35(1):79-86. http://dx.doi.org/10.1007/s11096-012-9710-7 PMID: 23161124

46. Neto PR, Marusic S, de Lyra Junior DP, Pilger D, Cruciol-Souza JM, Gaeti WP, et al. Effect of a 36-month pharmaceutical care program on the coronary heart disease risk in elderly diabetic and hypertensive patients. J Pharm Pharm Sci. 2011; 14(2):249-63. http://dx.doi.org/10.18433/j3259q PMID: 21733413

47. Paz-Pacheco E, Sandoval MA, Ardena GJ, Paterno E, Juban N, Lantion-Ang FL, et al. Effectiveness of a community-based diabetes self-management education (DSME) program in a rural agricultural setting. Prim Health Care Res Dev. 2017; 18(1):35-49. http://dx.doi.org/10.1017/S1463423616000335 PMID: 27640303

48. Phumipamorn S, Pongwecharak J, Soorapan S, Pattharachayakul S. Effects of the pharmacist's input on glycaemic control and cardiovascular risks in Muslim diabetes. Prim Care Diabetes. 2008; 2(1):31-7. http://dx.doi.org/10.1016/j.pcd.2007.12.001 PMID: 18684418

49. Prabhakaran D, Jha D, Prieto-Merino D, Roy A, Singh K, Ajay VS, et al. Effectiveness of an mHealth-Based Electronic Decision Support System for Integrated Management of Chronic Conditions in Primary Care: The mWellcare Cluster-Randomized Controlled Trial. Circulation. 2018. http://dx.doi.org/10.1161/CIRCULATIONAHA.118.038192 PMID: 30586732

50. Jha D, Gupta P, Ajay VS, Jindal D, Perel P, Prieto-Merino D, et al. Protocol for the mWellcare trial: a multicentre, cluster randomised, 12-month, controlled trial to compare the

effectiveness of mWellcare, an mHealth system for an integrated management of patients with hypertension and diabetes, versus enhanced usual care in India. BMJ Open. 2017; 7(8):e014851. http://dx.doi.org/10.1136/bmjopen-2016-014851 PMID: 28801393

51. Ramli AS, Selvarajah S, Daud MH, Haniff J, Abdul-Razak S, Tg-Abu-Bakar-Sidik TM, et al. Effectiveness of the EMPOWER-PAR Intervention in Improving Clinical Outcomes of Type 2 Diabetes Mellitus in Primary Care: A Pragmatic Cluster Randomised Controlled Trial. BMC Fam Pract. 2016; 17(1):157. http://dx.doi.org/10.1186/s12875-016-0557-1 PMID: 27842495

52. Ramli AS, Lakshmanan S, Haniff J, Selvarajah S, Tong SF, Bujang M-A, et al. Study protocol of EMPOWER Participatory Action Research (EMPOWER-PAR): a pragmatic cluster randomised controlled trial of multifaceted chronic disease management strategies to improve diabetes and hypertension outcomes in primary care. BMC Family Practice. 2014; 15(1):151. http://dx.doi.org/10.1186/1471-2296-15-151

53. Reutens AT, Hutchinson R, Van Binh T, Cockram C, Deerochanawong C, Ho LT, et al. The GIANT study, a cluster-randomised controlled trial of efficacy of education of doctors about type 2 diabetes mellitus management guidelines in primary care practice. Diabetes Res Clin Pract. 2012; 98(1):38-45. http://dx.doi.org/10.1016/j.diabres.2012.06.002 PMID: 22784926

54. Saleh S, Farah A, Dimassi H, El Arnaout N, Constantin J, Osman M, et al. Using Mobile Health to Enhance Outcomes of Noncommunicable Diseases Care in Rural Settings and Refugee Camps: Randomized Controlled Trial. JMIR Mhealth Uhealth. 2018; 6(7):e137. http://dx.doi.org/10.2196/mhealth.8146 PMID: 30006326

55. Sarayani A, Mashayekhi M, Nosrati M, Jahangard-Rafsanjani Z, Javadi M, Saadat N, et al. Efficacy of a telephone-based intervention among patients with type-2 diabetes; a randomized controlled trial in pharmacy practice. Int J Clin Pharm. 2018; 40(2):345-53. http://dx.doi.org/10.1007/s11096-018-0593-0 PMID: 29435911

56. Shen M, Zhao H, Sun K, Lu Y, Wang Z. Influence of pharmaceutical care intervention on type 2 diabetes patients with multi-drug therapy in the community. Pharmaceutical Care and Research. 2016; 16(3):170-4.

57. Sriram S, Chack LE, Ramasamy R, Ghasemi A, Ravi TK, Sabzghabaee AM. Impact of pharmaceutical care on quality of life in patients with type 2 diabetes mellitus. J Res Med Sci. 2011; 16 Suppl 1:S412-8. PMID: 22247727

58. Tutino GE, Yang WY, Li X, Li WH, Zhang YY, Guo XH, et al. A multicentre demonstration project to evaluate the effectiveness and acceptability of the web-based Joint Asia Diabetes Evaluation (JADE) programme with or without nurse support in Chinese patients with Type 2 diabetes. Diabet Med. 2017; 34(3):440-50. http://dx.doi.org/10.1111/dme.13164 PMID: 27278933

59. Van Olmen J, Kegels G, Korachais C, de Man J, Van Acker K, Kalobu JC, et al. The effect of text message support on diabetes self-management in developing countries - A

randomised trial. J Clin Transl Endocrinol. 2017; 7:33-41. http://dx.doi.org/10.1016/j.jcte.2016.12.005 PMID: 29067248

60. van Olmen J, Ku GM, van Pelt M, Kalobu JC, Hen H, Darras C, et al. The effectiveness of text messages support for diabetes self-management: protocol of the TEXT4DSM study in the democratic Republic of Congo, Cambodia and the Philippines. BMC Public Health. 2013; 13(1):423. http://dx.doi.org/10.1186/1471-2458-13-423

61. Van Olmen J, Marie KG, Christian D, Clovis KJ, Emery B, Maurits VP, et al. Content, participants and outcomes of three diabetes care programmes in three low and middle income countries. Prim Care Diabetes. 2015; 9(3):196-202. http://dx.doi.org/10.1016/j.pcd.2014.09.001 PMID: 25281167

62. Van Olmen J, Van Pelt M, Malombo B, Ku GM, Kanda D, Heang H, et al. Process evaluation of a mobile health intervention for people with diabetes in low income countries - the implementation of the TEXT4DSM study. J Telemed Telecare. 2017; 23(1):96-105. http://dx.doi.org/10.1177/1357633X15617885 PMID: 26656894

63. Wang L, Fang H, Xia Q, Liu X, Chen Y, Zhou P, et al. Health literacy and exercise-focused interventions on clinical measurements in Chinese diabetes patients: A cluster randomized controlled trial. EClinicalMedicine. 2019; 17:100211. http://dx.doi.org/10.1016/j.eclinm.2019.11.004 PMID: 31891144

64. Xu WH, Rothman RL, Li R, Chen Y, Xia Q, Fang H, et al. Improved self-management skills in Chinese diabetes patients through a comprehensive health literacy strategy: study protocol of a cluster randomized controlled trial. Trials. 2014; 15(1):498. http://dx.doi.org/10.1186/1745-6215-15-498

65. Liu XN, Xia QH, Fang H, Li R, Chen YY, Yan YJ, et al. [Effect of health literacy and exercise-focused interventions on glycemic control in patients with type 2 diabetes in China]. Zhonghua Liu Xing Bing Xue Za Zhi. 2018; 39(3):357-62. http://dx.doi.org/10.3760/cma.j.issn.0254-6450.2018.03.021 PMID: 29609254

66. Wishah RA, Al-Khawaldeh OA, Albsoul AM. Impact of pharmaceutical care interventions on glycemic control and other health-related clinical outcomes in patients with type 2 diabetes: Randomized controlled trial. Diabetes Metab Syndr. 2015; 9(4):271-6. http://dx.doi.org/10.1016/j.dsx.2014.09.001 PMID: 25301007

67. Zhong X, Wang Z, Fisher EB, Tanasugarn C. Peer Support for Diabetes Management in Primary Care and Community Settings in Anhui Province, China. Ann Fam Med. 2015; 13 Suppl 1:S50-8. http://dx.doi.org/10.1370/afm.1799 PMID: 26304972
