# Supplementary material for: Health system interventions for adults with type 2 diabetes in low- and middle-income countries: A systematic review and meta-analysis
Source: PLoS Med. 2020 Nov 12;17(11):e1003434. doi: 10.1371/journal.pmed.1003434 (PMC7660583; doi:10.1371/journal.pmed.1003434)
Supplement: S6 Appendix — (PDF) [file pmed.1003434.s006.pdf]

S6 Appendix: Forest plot of overall deaths by study

Study	Treatment Yes	Treatment No	Control Yes	Control No	Odds Ratio with 95% CI	Weight (%)
**Multicomponent clinic–based intervention**						
Ali	12	471	14	460	0.84 (0.38, 1.83)	10.84
Anzaldo–Campos	0	82	0	92	1.12 (0.02, 57.14)	0.43
Chao	0	50	0	50	1.00 (0.02, 51.38)	0.43
Khan	0	232	0	218	0.94 (0.02, 47.57)	0.43
Prabhakaran	34	902	21	903	1.62 (0.93, 2.81)	21.75
Ramli	6	465	3	414	1.78 (0.44, 7.16)	3.42
**Pharmacist task sharing**						
Ayadurai	0	49	1	62	0.42 (0.02, 10.56)	0.64
Javaid	0	83	1	51	0.21 (0.01, 5.14)	0.64
Shen	0	73	2	73	0.20 (0.01, 4.24)	0.71
Sriram	0	60	0	60	1.00 (0.02, 51.22)	0.43
**Diabetes education or support alone**						
Guo	5	70	3	65	1.55 (0.36, 6.74)	3.06
Paz–Pacheco	2	70	1	51	1.46 (0.13, 16.51)	1.12
Van Olmen	30	371	36	344	0.77 (0.47, 1.28)	25.84
**Case management by nurses**						
DePue	2	146	2	93	0.64 (0.09, 4.60)	1.69
Tutino	6	1,377	6	1,170	0.85 (0.27, 2.64)	5.15
**Physician clinical training alone**						
Reutens	0	199	1	186	0.31 (0.01, 7.70)	0.64
**Multicomponent nurse task sharing**						
Fairall	30	131	31	187	1.38 (0.80, 2.39)	21.94
**Internet–based glucose telemonitoring**						
Kim	0	92	0	90	0.98 (0.02, 49.84)	0.43
Lee	0	104	0	104	1.00 (0.02, 50.87)	0.43

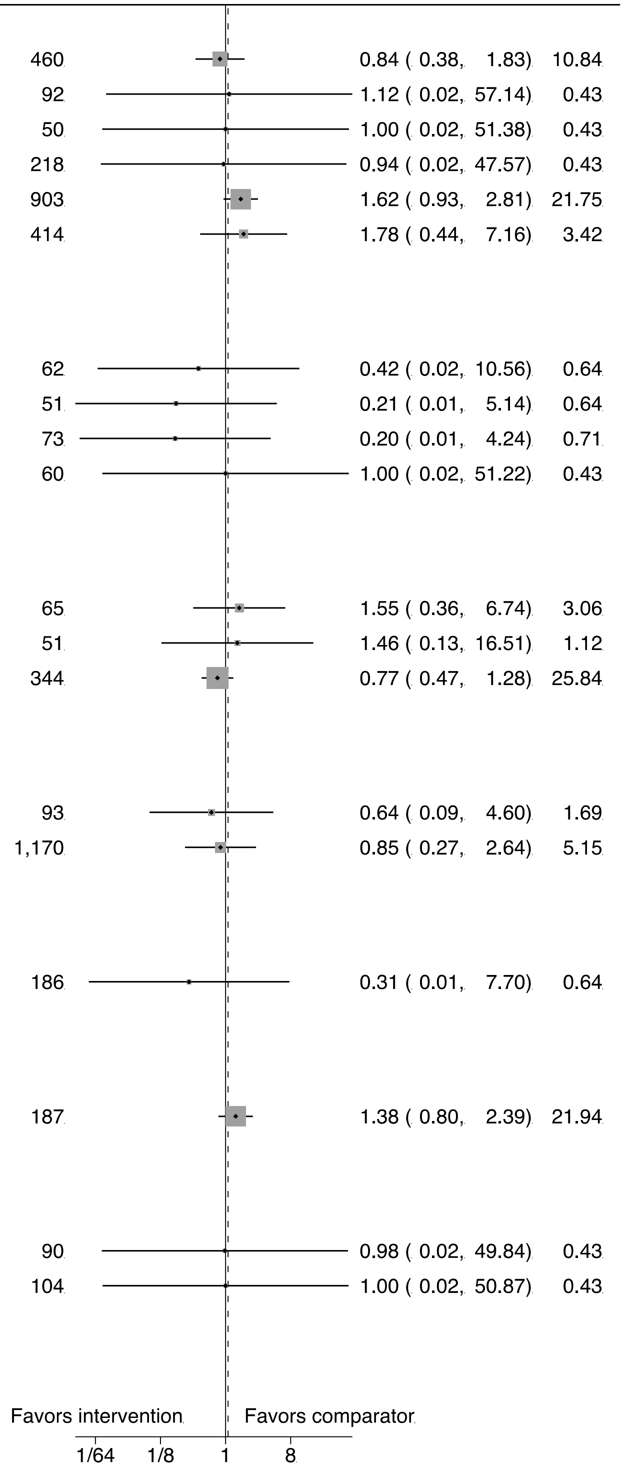

Note: Summary estimates of mortality have been suppressed due to sparseness of data. Deaths are indicated by the “Yes” column.
