# Supplementary material for: Health system interventions for adults with type 2 diabetes in low- and middle-income countries: A systematic review and meta-analysis
Source: PLoS Med. 2020 Nov 12;17(11):e1003434. doi: 10.1371/journal.pmed.1003434 (PMC7660583; doi:10.1371/journal.pmed.1003434)
Supplement: S7 Appendix — (PDF) [file pmed.1003434.s007.pdf]

S7 Appendix: EPOC Risk of bias assessment

Author and year	Random sequence generation	Allocation concealment	Baseline outcome measurements similar	Baseline characteristics similar	Incomplete outcome data	Knowledge of the allocated interventions adequately prevented during the study	Protection against contamination	Selective outcome reporting	Other risks of bias	Summary risk of bias
Adibe 2013	Low	Unclear	Low	Low	Low	Low	Unclear	Low	Low	Unclear
Akturan 2017	Unclear	Unclear	Low	Low	Unclear	Low	Unclear	Low	High	High
Ali 2016	Low	Low	Low	Low	Low	Unclear	Unclear	Low	Low	Unclear
Anzaldo-Campos 2016	Unclear	Unclear	Low	Low	Low	Unclear	Low	Low	Low	Unclear
Ayadurai 2018	Low	Low	Low	Low	High	Low	Low	Low	Low	High
Barcelo 2010	Unclear	Low	Low	Low	Unclear	Low	Low	Low	Unclear	Unclear
Chao 2015	Low	Low	Low	Low	Low	Low	High	Low	Low	High
Chapman 2018	Low	Low	Low	Low	Low	Low	Low	Low	Low	Low
Chung 2014	Unclear	Unclear	Low	Low	Unclear	Low	High	Low	Low	High
DePue 2013	Unclear	Low	Low	Low	Low	Low	Low	Low	Low	Unclear
Fairall 2016	Low	Low	Low	Low	Low	Low	Low	Low	Low	Low
Gillani 2016	Unclear	Unclear	Low	Low	Unclear	Low	Unclear	Low	Unclear	Unclear
Goruntla 2019	Unclear	Unclear	Low	Low	Low	Low	High	Low	Low	High
Guo 2019	Low	Low	Low	Low	Low	Low	High	Low	Low	High
Jaipakdee 2015	Unclear	Unclear	Low	Low	Unclear	Low	Low	Low	Low	Unclear
Jarab 2012	Low	Low	Low	Low	Low	Low	High	Low	Low	High
Javaid 2019	Low	Low	Low	High	High	Low	High	Low	Low	High
Khan 2018	Low	Low	Low	Low	Low	Low	Low	Low	Low	Low
Khetan 2019	Low	Low	Low	Low	Low	Low	Low	Low	Low	Low
Kim 2016	Unclear	Unclear	Low	Low	Low	Low	High	Low	Unclear	High
Kong 2019	Unclear	Unclear	Low	Unclear	Low	Low	Low	Low	Unclear	Unclear
Lee 2020	Low	Low	Low	Low	Low	Low	Low	Low	Low	Low
Mash 2014	Low	Low	Low	Low	Low	Low	Low	Low	Low	Low
Mourao 2013	Low	Unclear	Low	Low	Low	Low	Unclear	Low	Low	Unclear
Neto 2011	Low	Unclear	Low	Low	Low	Low	High	Low	Low	High

Paz-Pacheco 2017	Unclear	Unclear	Low	Low	Unclear	Low	Low	Low	Low	Unclear
Phumipamorn 2008	Low	Low	Low	Low	Low	Low	High	Low	Low	High
Prabhakaran 2018	Low	Low	Low	Low	Low	Low	Low	Low	Low	Low
Ramli 2016	Low	Low	Low	Low	Low	Low	Low	Low	Low	Low
Reutens 2012	Low	Unclear	Low	Low	Low	Low	Low	Low	Low	Unclear
Saleh 2018	Unclear	Low	Low	Low	Unclear	Low	Low	Low	Unclear	Unclear
Sarayani 2018	Unclear	Low	Low	Low	Low	Low	Unclear	Low	Low	Unclear
Shen 2016	Unclear	Low	Low	Low	Low	Unclear	Low	Low	Low	Unclear
Sriram 2011	Unclear	Unclear	Low	Unclear	Unclear	Unclear	High	Unclear	Low	High
Tutino 2017	Low	Low	Low	Low	Low	Low	Unclear	Low	Low	Unclear
VanOlmen 2017	Unclear	Low	Low	Low	Low	Low	High	Low	Unclear	High
Wang 2019	Unclear	Low	Low	High	Low	Low	Low	Low	Low	High
Wishah 2015	Low	Low	High	Unclear	Low	Low	High	Low	Low	High
Zhong 2015	Unclear	Unclear	Unclear	High	High	Low	Low	Low	Low	High
