# Supplementary figures and images for: Health system interventions for adults with type 2 diabetes in low- and middle-income countries: A systematic review and meta-analysis
Source: PLoS Med. 2020 Nov 12;17(11):e1003434. doi: 10.1371/journal.pmed.1003434 (PMC7660583; doi:10.1371/journal.pmed.1003434)

**S8 Appendix: Overall funnel plot (all studies)**

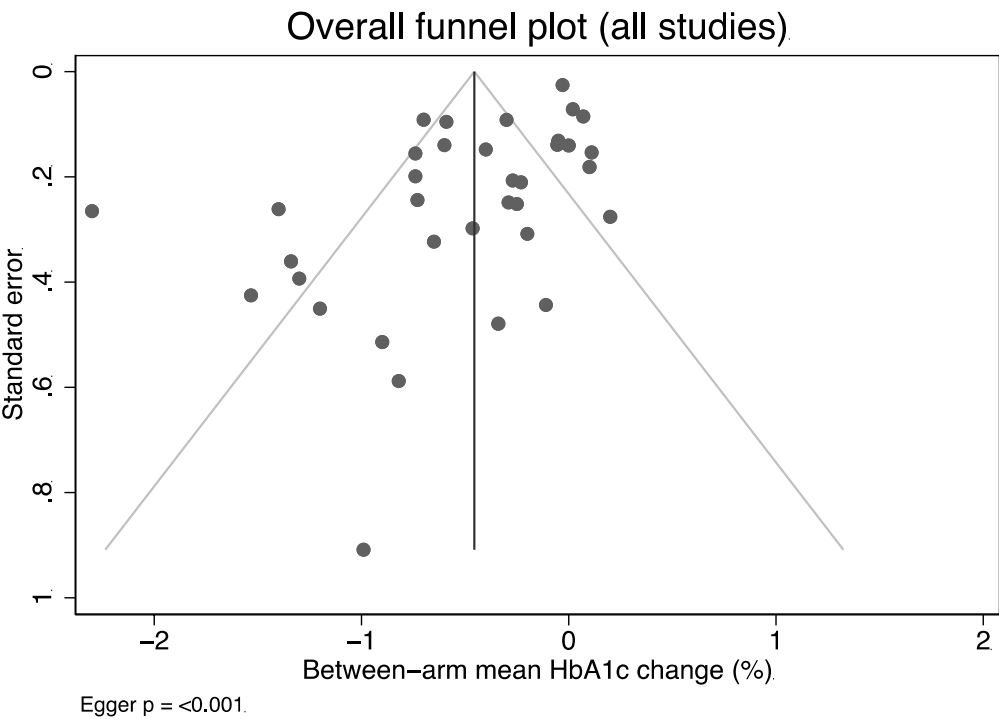

Supplement: S8 Appendix — (PDF) [file pmed.1003434.s008.pdf]

S9 Appendix: Funnel plot by intervention type (all studies)

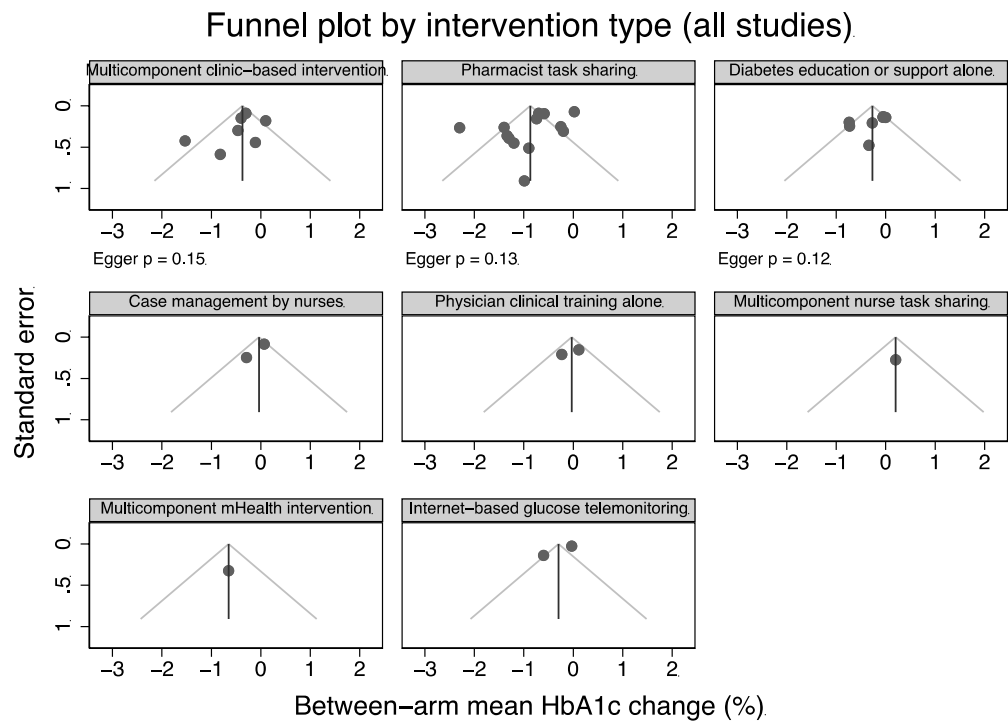

Supplement: S9 Appendix — (PDF) [file pmed.1003434.s009.pdf]

S10 Appendix: Funnel plot generated using trim-and-fill method

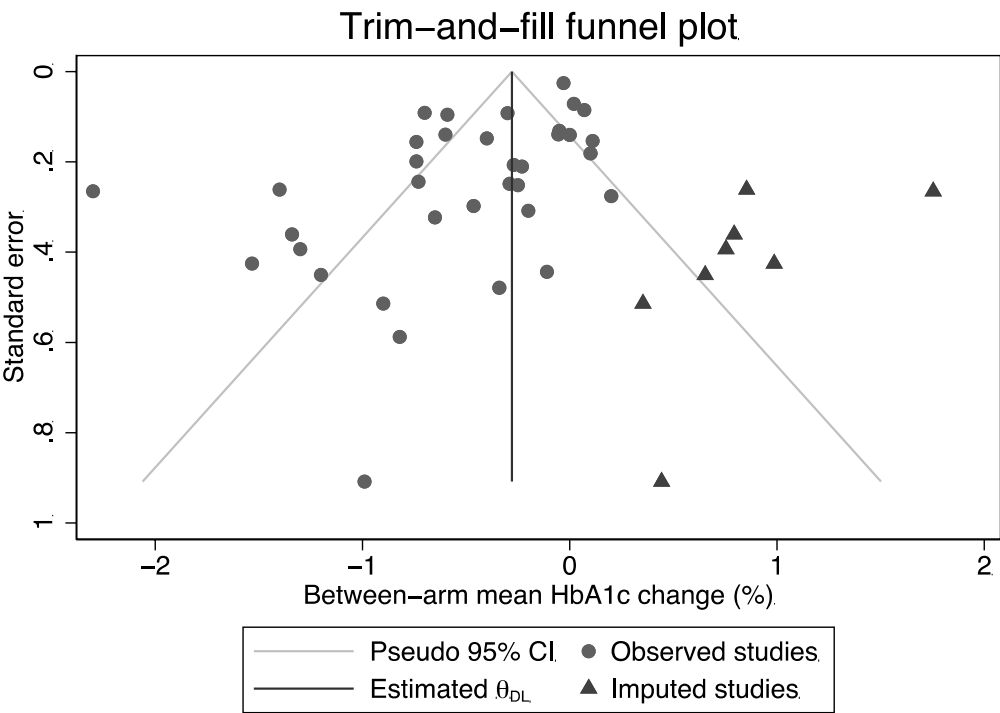

Supplement: S10 Appendix — (PDF) [file pmed.1003434.s010.pdf]

**S12 Appendix: Overall funnel plot (excluding studies at high risk of bias)**

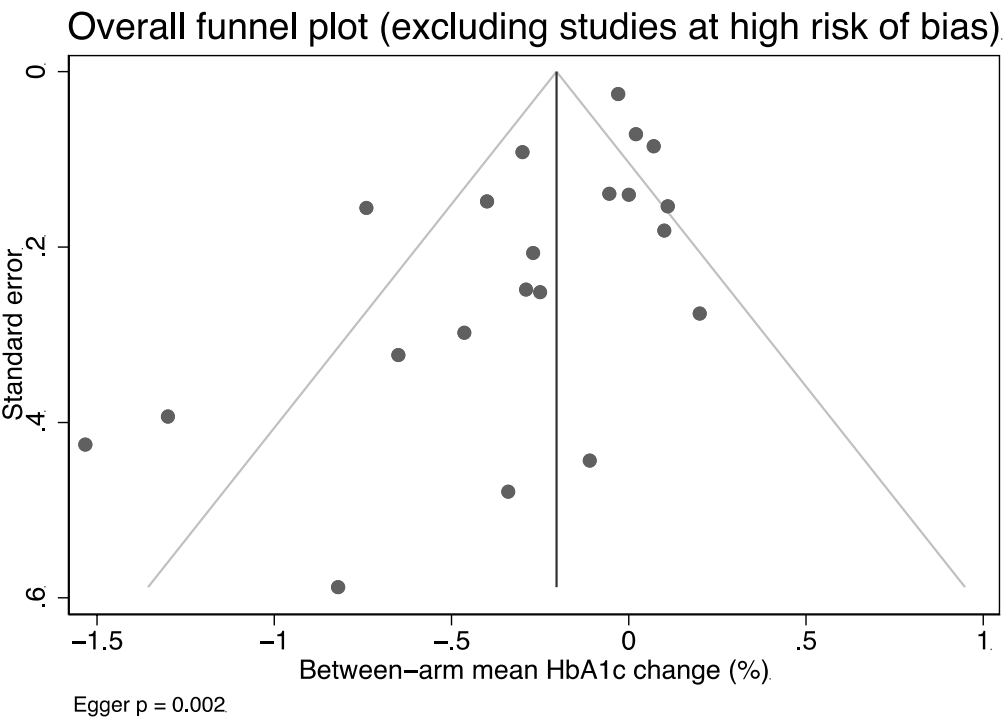

Supplement: S12 Appendix — (PDF) [file pmed.1003434.s012.pdf]

S13 Appendix: Funnel plot by intervention type (excluding studies at high risk of bias)

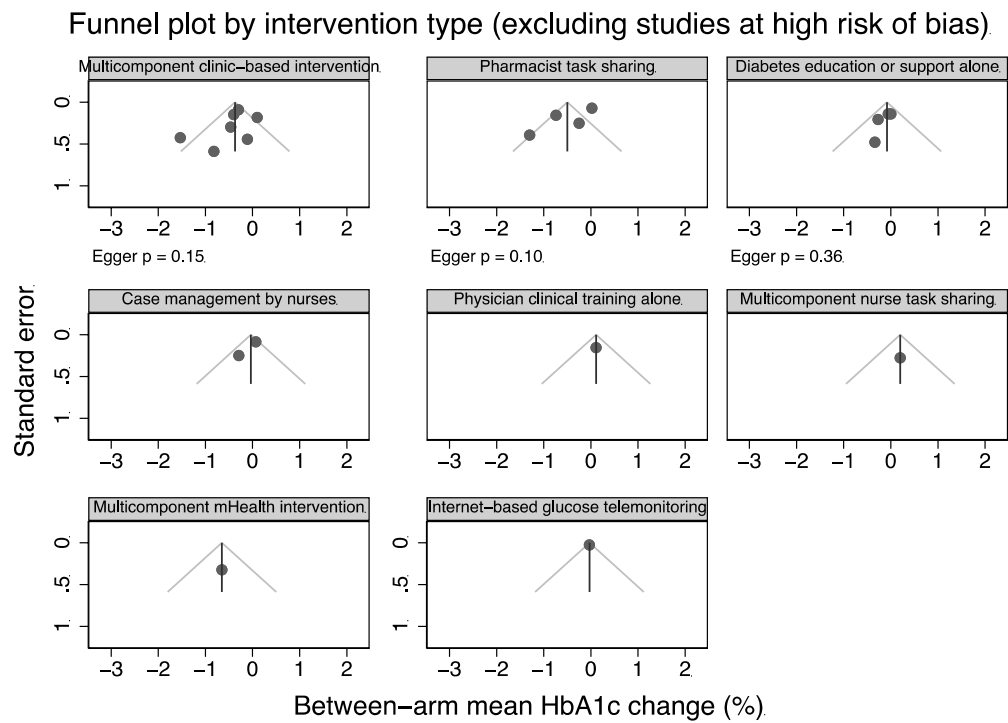

Supplement: S13 Appendix — (PDF) [file pmed.1003434.s013.pdf]
