# Supplementary material for: Health system interventions for adults with type 2 diabetes in low- and middle-income countries: A systematic review and meta-analysis
Source: PLoS Med. 2020 Nov 12;17(11):e1003434. doi: 10.1371/journal.pmed.1003434 (PMC7660583; doi:10.1371/journal.pmed.1003434)

S11 Appendix: Forest plot for meta-analysis of HbA1c (%) mean difference (excluding studies at high risk of bias)

Study	Treatment N	Treatment Mean	Treatment SD	Control N	Control Mean	Control SD	Mean Diff. with 95% CI	Weight (%)
**Multicomponent clinic–based intervention**								
Ali	483	−1.60	2.24	474	−1.20	2.33	−0.40 (−0.69, −0.11)	6.26
Anzaldo–Campos	171	−2.83	3.29	92	−1.30	3.29	−1.53 (−2.37, −0.70)	1.71
Barcelo	63	−0.52	1.54	36	−0.05	1.20	−0.46 (−1.05, 0.12)	2.96
Khan	94	−2.26	3.61	88	−1.44	4.31	−0.82 (−1.97, 0.33)	0.98
Kong	129	−0.57	1.92	119	−0.46	4.62	−0.11 (−0.98, 0.76)	1.60
Prabhakaran	331	−0.48	2.32	326	−0.58	2.32	0.10 (−0.25, 0.45)	5.29
Ramli	83	−0.10	0.55	74	0.20	0.60	−0.30 (−0.48, −0.12)	8.07
Heterogeneity: $\tau^2 = 0.06$, $I^2 = 60.00\%$, $H^2 = 2.50$							−0.37 (−0.64, −0.10)	
**Pharmacist task sharing**								
Adibe	99	−0.75	1.08	93	−0.02	1.08	−0.74 (−1.04, −0.44)	6.03
Mourao	50	−0.60	1.95	50	0.70	1.98	−1.30 (−2.07, −0.53)	1.95
Sarayani	40	−0.88	0.33	44	−0.90	0.33	0.02 (−0.12, 0.16)	8.68
Shen	42	−0.42	1.27	43	−0.17	1.04	−0.25 (−0.74, 0.24)	3.70
Heterogeneity: $\tau^2 = 0.25$, $I^2 = 89.58\%$, $H^2 = 9.60$							−0.50 (−1.04, 0.04)	
**Diabetes education or support alone**								
Chapman	55	−3.65	1.04	44	−3.38	1.00	−0.27 (−0.68, 0.14)	4.64
Jaipakdee	67	−0.38	0.61	63	−0.32	0.95	−0.05 (−0.33, 0.22)	6.53
Mash	186	−0.50	1.42	226	−0.50	1.42	0.00 (−0.27, 0.27)	6.49
Paz–Pacheco	53	−0.30	2.33	38	0.04	2.13	−0.34 (−1.28, 0.60)	1.40
Heterogeneity: $\tau^2 = 0.00$, $I^2 = 0.00\%$, $H^2 = 1.00$							−0.08 (−0.25, 0.09)	
**Case management by nurses**								
DePue	64	−0.28	1.45	41	0.01	0.82	−0.29 (−0.78, 0.20)	3.76
Tutino	1,383	−0.62	2.18	1,176	−0.69	2.10	0.07 (−0.10, 0.24)	8.27
Heterogeneity: $\tau^2 = 0.03$, $I^2 = 46.77\%$, $H^2 = 1.88$							−0.03 (−0.35, 0.29)	
**Physician clinical training alone**								
Reutens	111	−0.17	1.11	104	−0.28	1.14	0.11 (−0.19, 0.41)	6.08
Heterogeneity: $\tau^2 = 0.00$, $I^2 = .\%$, $H^2 = .$							0.11 (−0.19, 0.41)	
**Multicomponent nurse task sharing**								
Fairall	114	0.00	2.40	154	−0.20	2.10	0.20 (−0.34, 0.74)	3.29
Heterogeneity: $\tau^2 = 0.00$, $I^2 = .\%$, $H^2 = .$							0.20 (−0.34, 0.74)	
**Multicomponent mHealth intervention**								
Saleh	115	−0.87	2.04	67	−0.22	2.21	−0.65 (−1.28, −0.02)	2.63
Heterogeneity: $\tau^2 = 0.00$, $I^2 = .\%$, $H^2 = .$							−0.65 (−1.28, −0.02)	
**Internet–based glucose telemonitoring**								
Lee	52	−0.33	0.15	52	−0.30	0.11	−0.03 (−0.08, 0.02)	9.68
Heterogeneity: $\tau^2 = 0.00$, $I^2 = .\%$, $H^2 = .$							−0.03 (−0.08, 0.02)	
**Overall**							−0.20 (−0.32, −0.08)	
Heterogeneity: $\tau^2 = 0.04$, $I^2 = 71.76\%$, $H^2 = 3.54$								

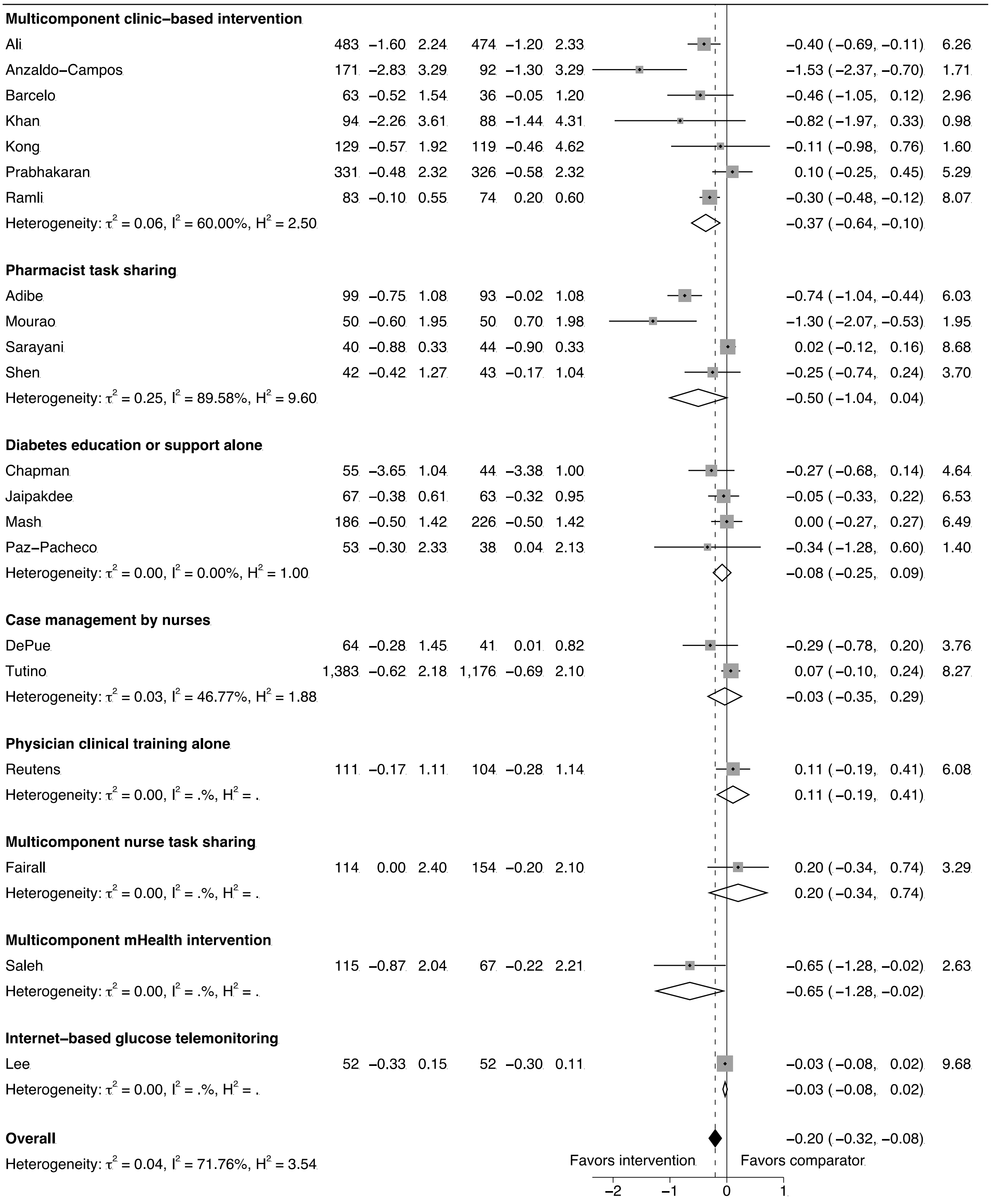

Supplement: S11 Appendix — (PDF) [file pmed.1003434.s011.pdf]
