# Supplementary material for: Health system interventions for adults with type 2 diabetes in low- and middle-income countries: A systematic review and meta-analysis
Source: PLoS Med. 2020 Nov 12;17(11):e1003434. doi: 10.1371/journal.pmed.1003434 (PMC7660583; doi:10.1371/journal.pmed.1003434)
Supplement: S14 Appendix — (PDF) [file pmed.1003434.s014.pdf]

S14 Appendix: Forest plot generated using leave-one-out method

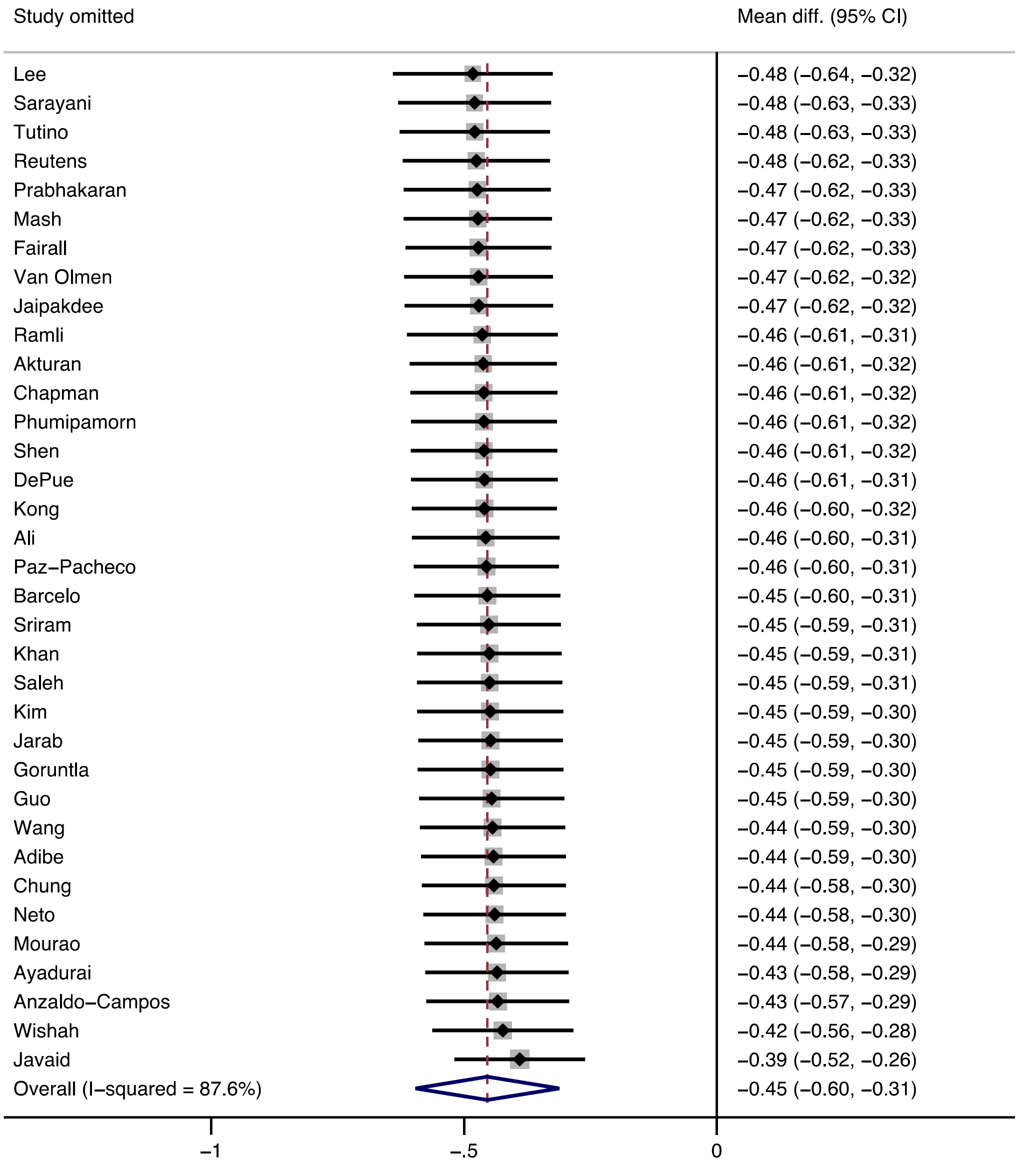

NOTE: Weights are from random−effects model
