# Supplementary material for: Health system interventions for adults with type 2 diabetes in low- and middle-income countries: A systematic review and meta-analysis
Source: PLoS Med. 2020 Nov 12;17(11):e1003434. doi: 10.1371/journal.pmed.1003434 (PMC7660583; doi:10.1371/journal.pmed.1003434)
Supplement: S15 Appendix — (PDF) [file pmed.1003434.s015.pdf]

Health systems interventions compared to usual care for community-dwelling, ambulatory adults with type 2 diabetes in low- and middle-income countries

Patient or population: Community-dwelling, ambulatory adults with type 2 diabetes
Setting: Low- and middle-`income countries
Intervention: Health systems interventions
Comparison: Usual care

Outcomes	Hemoglobin A1c mean difference (95% CI)	Number of participants (studies)*	Certainty of the evidence (GRADE)	Results in words
Glycemic change from baseline (Multicomponent clinic based)	**-0.46%** (-0.64 to -0.10)	5,397† (8 RCTs)	⊕⊕⊕◯ Moderate	Multicomponent clinic-based interventions probably lead to modest improvements in glycemic control.
Glycemic change from baseline (Pharmacist task sharing)	**-0.87%** (-1.20 to -0.53)	2,331 (14 RCTs)	⊕⊕◯◯ Low	Pharmacist task sharing interventions may improve glycemic control.
Glycemic change from baseline (Diabetes education or support alone)	**-0.27%** (-0.50 to -0.04)	5,924† (9 RCTs)	⊕⊕◯◯ Low	Diabetes education or support alone interventions may slightly improve glycemic control.
Glycemic change from baseline (Case management by nurses)	**-0.03%** (-0.35 to 0.29)	3,854 (2 RCTs)	⊕◯◯◯ Very low	It is uncertain whether case management interventions by nurses improve glycemic control because the certainty of the evidence is very low.
Glycemic change from baseline (physician clinical training alone)	**-0.03%** (-0.36 to 0.30)	498 (2 RCTs)	⊕◯◯◯ Very low	It is uncertain whether physician training interventions improve clinical care improves glycemic control because the certainty of the evidence is very low.
Glycemic change from baseline (Nurse task sharing)	**0.20%** (-0.34 to 0.74)	1,842 (1 RCTs)	⊕⊕◯◯ Low	Nurse task sharing interventions may lead to little or no difference in glycemic control.
Glycemic change from baseline (mHealth screening and quality improvement)	**-0.65%** (-1.28 to -0.02)	812 (1 RCTs)	⊕◯◯◯ Very low	It is uncertain whether mHealth interventions improve glycemic control because the certainty of the evidence is very low.
Glycemic change from baseline (Internet-based glucose telemonitoring)	**-0.30%** (-0.86 to 0.26)	422 (2 RCTs)	⊕◯◯◯ Very low	It is uncertain whether internet-based glucose telemonitoring interventions improve glycemic control because the certainty of the evidence is very low.

*Refers to total participants not including adjustments made for sample sizes for cluster RCTs were adjusted to account for the design effect.
†Includes participants in trials in which glycemic change was assessed as fasting glucose rather than HbA1c. These trials were not incorporated in the meta-analysis of HbA1c but are considered in the Summary of Findings.

CI: Confidence interval

GRADE Working Group grades of evidence
High = This research provides a very good indication of the likely effect. The likelihood that the effect will be substantially different† is low.
Moderate = This research provides a good indication of the likely effect. The likelihood that the effect will be substantially different† is moderate.
Low = This research provides some indication of the likely effect. However, the likelihood that it will be substantially different† is high.
Very low = This research does not provide a reliable indication of the likely effect. The likelihood that the effect will be substantially different† is very high.
† Substantially different = a large enough difference that it might affect a decision
