# Supplementary material for: Health system interventions for adults with type 2 diabetes in low- and middle-income countries: A systematic review and meta-analysis
Source: PLoS Med. 2020 Nov 12;17(11):e1003434. doi: 10.1371/journal.pmed.1003434 (PMC7660583; doi:10.1371/journal.pmed.1003434)
Supplement: S16 Appendix — (PDF) [file pmed.1003434.s016.pdf]

S16 Appendix: Certainty assessment of evidence the glycemic control

Comparison: Usual care

Study category	No of studies	Design	Risk of bias	Inconsistency	Indirectness	Imprecision	Other	Certainty (overall score)
Multicomponent clinic-based interventions	8[a]	4	0	-0.5[b]	-1[c]	0	+0.5[d]	3
Pharmacist task sharing	14[e]	4	-2[f]	0	0	0	0	2
Diabetes education or support alone	9[g]	4	0	-1[h]	0	0	-1[i]	2
Case management by nurses	2	4	-1[j]	-1[k]	-1[l]		0	1
Physician training to improve clinical care	2	4	-1[m]	-1[n]	-1[o]	0	0	1
Multicomponent nurse task sharing	1	4	0	-2[p]	0	0	0	2
Multicomponent mHealth	1	4	-1[q]	-2	0	0	0	1
Internet-based glucose telemonitoring	2	4	-1[r]	-2[s]	0	0	0	1

Detailed instructions on the methodology used to generate the certainty of evidence can be found at the following citation:

Cochrane Effective Practice and Organisation of Care (EPOC). EPOC worksheets for preparing a Summary of Findings (SoF) table using GRADE. EPOC Resources for review authors, 2017. Available at: http://epoc.cochrane.org/resources/epoc-resources-review-authors

[a] One study (Chao) reported fasting glucose and not HbA1c. This study was not included in the meta-analysis of HbA1c
[b] Two very well-conducted studies with low risk of bias (Prabhakaran and Khan) were null.
[c] There are relatively large differences in intervention components and the populations to which they were applied.
[d] Two studies (Prabhakaran and Khan) compared the intervention to enhanced usual care, which consisted of additional resources directed to the control group. This represents a plausible factor that would reduce the demonstrated effect of this interventions.
[e] One study reported HbA1c but did not report uncertainty estimates so was not included in the meta-analysis.
[f] Nine of the 14 pharmacist-led studies had high summary risk of bias for the outcome of glycemic control due to inadequate protection against contamination, differences in baseline outcomes, and other risks. The remaining 5 studies had unclear summary risk. No pharmacist-led interventions had low risk of bias.
[g] Two studies (Zhong and Khetan) reported fasting glucose and not HbA1c. These two studies were not included in the meta-analysis.
[h] All three studies with low risk of bias were null (Chapman, Khetan, Mash).
[i] The sizeable within-group decrease in mean HgA1c in comparator arms of some studies represents a plausible factor that would reduce the demonstrated effect of this intervention and may lead to negative trial results.
[j] The two available studies had unclear risk of bias.
[k] There were inconsistent results in two the two available studies..
[l] There were considerable differences in intervention details and population.
[m] There were only two studies in this category, one of which had unclear risk of bias and one low risk of bias.
[n] Of the two studies, one was a null trial.
[o] Considerable difference in intervention details and population
[p] Only a single null trial (Fairall), though low risk of bias.
[q] Only a single study (Saleh) at unclear risk of bias.
[r] Only two studies, one unclear and one low risk of bias.
[s] Inconsistent results in two trials.
