# Supplementary material for: Health system interventions for adults with type 2 diabetes in low- and middle-income countries: A systematic review and meta-analysis
Source: PLoS Med. 2020 Nov 12;17(11):e1003434. doi: 10.1371/journal.pmed.1003434 (PMC7660583; doi:10.1371/journal.pmed.1003434)
Supplement: Attachment — Submitted filename: 2020_07_28 - Responses to reviewers - Flood review.docx [file pmed.1003434.s017.docx]

**Health system interventions for adults with type 2 diabetes in low- and middle-income countries: A systematic review and meta-analysis**

**Responses to Reviewers**

**July 21, 2020**

-----------------------------------------------------------

**Requests from the editors:**

***In the last sentence of the Abstract Methods and Findings section, please add a brief note summarising any key limitation(s) of the study's methodology.**

We have added a sentence in the Abstract “Methods and Findings” section (bottom of page 3) with this information.

***At this stage, we ask that you include a short, non-technical Author Summary of your research to make findings accessible to a wide audience that includes both scientists and non-scientists. The Author Summary should immediately follow the Abstract in your revised manuscript. This text is subject to editorial change and should be distinct from the scientific abstract. Please see our author guidelines for more information:** [**https://journals.plos.org/plosmedicine/s/revising-your-manuscript#loc-author-summary**](https://journals.plos.org/plosmedicine/s/revising-your-manuscript#loc-author-summary)

We have included a short, non-technical Author Summary on pages 5-6.

-----------------------------------------------------------

**Reviewer #1:**

**This is an important but challenging area to investigate. The authors have done an excellent job of bringing together a large number of trials. The challenge is in the interpretation of findings when the results of studies are grouped and meta-analysed. The large I-squared values indicate the statistical risks in pooling these studies. This is compounded by the obvious heterogeneity of study design. Even within the broad categories of study type, there are unlikely to be two studies whose description of their intervention was the same, never mind how and how successfully the protocol was implemented. Added to this is the variation in the setting and populations, which would likely impact on the potential for and size of any benefit. Thus, it is quite likely that within the studies in each group there are interventions that work and some that don't. Indeed, it is likely that the need for some studies was justified on the basis that they were attempting to improve on other similar interventions that failed. In such circumstances it is very hard to know what a pooled estimate means, or even how to interpret general comments about the benefits of, for example, multicomponent interventions. It seems that it would be more useful to try to understand what features characterized the studies with the larger effect sizes. Of course this is also inherently difficult, as conclusions may then be driven by very small numbers of studies. Ultimately, it seems that with current methodologies, it is very hard to make robust conclusions in this area.**

We thank Reviewer #1 for their view that we did an excellent job bringing together a large number of trials.

We agree that this is a challenging area to investigate. In the context of the extraordinary burden of diabetes worldwide, we think it is important to try to meet this challenge. We think our review adds value in a few ways:

- We have mapped the literature on health systems intervention in low- and middle-income countries and pointed out important gaps for future research. For example, only 1 trial was conducted in a site in a low-income country, and only 5 trials were primarily conducted in a rural setting. Additionally, several high-quality studies with supporting evidence from high-income countries—including all three diabetes education or support trials with low risk of bias—were null.
- A pooled estimate does not tell the whole story for health systems interventions, but there are substantial similarities within interventions types. Our authorship team, which includes several implementers of diabetes interventions, has discussed this issue extensively. We believe a meta-analysis is reasonable for the outcome of HbA1c. The heterogeneity in the pooled estimate, in fact, adds valuable data regarding just how variable these programs are with respect to a hard outcome. Together with our detailed narrative review, we believe this will help inform future work and future research in this area.
- That being said, our paper does not rely solely on pooled estimates or statistical measures of heterogeneity. As Reviewer #1 suggests, we have attempted to carefully describe the different components of each intervention type, drawing comparisons and contrasts when relevant. We also have highlighted studies with low risk of bias through the results and discussion section, as well as incorporating these assessments into our “Summary of Findings” table (S12 Appendix).
- We have added the following sentence in the discussion (page 23, line 389) to further highlight Reviewer #1’s point about the difficulty of making robust conclusions in this area: “Finally, although there were substantial similarities within intervention types, individual studies varied by setting and population, limiting our ability to make conclusions with high degrees of certainty..”

-----------------------------------------------------------

**Reviewer #2: See attachment**

**This paper describes a systematic review with meta–analysis of studies of interventions for Type 2 diabetes in low and middle income countries. I was asked for a statistical report and I interpret that to include all aspects of the design and conduct of the study.**

**Points of detail**

**Page 7 Page 6 told us that any language was accepted but here in the inclusion criteria we find that only English language articles were included.**

Our searches were run without language restrictions in the bibliographic databases. However, we only included studies that were published in English. To clarify this issue for the reader, we have removed the sentence referring to the search language (now page 8, line 46) that previously read: “All searches were run without limits or language restrictions.”

We have maintained the following sentence that details language inclusion criteria (page 9, line 54): “Included studies were published in English and reported at least one of the following outcomes: glycemic changes, mortality, health-related quality of life, or cost-effectiveness.”

We also discuss language inclusion in our discussion as a limitation of our study (page 23, line 387): “Consistent with similar prior reviews [7, 66], our review was restricted to studies in English with at least 6 months of follow-up and 100 enrolled participants.”

**Does this perhaps mean that as a matter of fact although studies in other languages were searched for none was found? In that case it should be in the results not the methods.**

We describe in “Fig 1: PRISMA study flow diagram” that of the 322 full-text articles reviewed, 284 articles were excluded including 20 articles with non-English language of publication. In the manuscript’s results section, we have highlighted the two most common reasons for full-text exclusion (type of intervention and incomplete data; page 11, line 113).

**Page 8 I did not understand the sentence starting ‘We followed the methodology’. I think a brief clarification of what the method is would help. Unfortunately I do not understand what incomplete HbA1c results means either which might have clarified the method being used.**

Please forgive us if this sentence was not clear (now bottom of page 10, line 93). We were referring to the variability that we observed in the way that studies reported HbA1c statistics. For example, some studies reported within-group changes from baseline as confidence intervals, standard errors, or p-values. Other studies only reported between-group statistics. Still others only reported baseline and endpoint HbA1c mean and test statistics.

We used the algebraic relationships outlined in section 7.7 of the 2011 Cochrane Handbook to calculate the within-group standard deviations when this information was not directly reported in the study and was not provided by authors when we inquired on two separate occasions.

We have modified the sentence Reviewer #2 refers to as follows:

“We followed the methodology recommended in the Cochrane handbook to calculate within-group mean and standard deviation when this information was not directly reported in the study or made available by authors [17].”

If Reviewer #2 believes that this sentence adds unnecessary ambiguity, we would be happy to omit it.

**Page 9 Confidence intervals for I2 would also be helpful to reveal our uncertainty about the heterogeneity. I know we did not use to use them but I am persuaded by Ioannidis et al. (2007) that we should. For some of the meta–analyses I think the authors might be surprised at how uncertain our estimates of I2 are. To be fair the authors do not put much stress on their estimates of I2.**

We now report 95% confidence intervals for I^2^ in the manuscript and main figures. We have added the following sentence in the methods (page 11, line 98) describing our use of these confidence intervals:

“Heterogeneity was explored by calculating I^2^ and T^2^, and we report 95% confidence intervals for I^2^ if three or more studies are pooled [19].”

Reference [19] refers to the following meta-analysis reference text, which we used to calculate the 95% confidence intervals for I^2^:

Borenstein M, Hedges LV, Higgins JP, Rothstein HR. Introduction to Meta-Analysis. West Sussex, United Kingdom: John Wiley & Sons; 2009.

**Page 14 The authors report on page 3 that outcomes of mortality, health–related quality of life (HQoL), and cost–effectiveness were infrequently reported but here we learn there were 18, 11, and 5. I can understand 5 being infrequent but in many reviews 18 would be seen as luxurious.**

- **I would have thought reporting of mortality would be beneficial. If it is sparse data at least report it even if the authors do not wish to perform a formal meta–analysis. If they do then Bradburn et al.(2007) may be helpful. If you have frequencies in each arm for 18 studies then the statement on page 20 that HbA1C was the only outcome which could be meta–analysed is incorrect.**

We have revised the description of mortality outcomes on page 16, line 212 based on Reviewer #2’s comments. In general, we do not wish to pursue a meta-analysis for mortality as the data is very sparse and offers limited statistical power to pool data. We have made the following revisions:

- We have clarified that full outcome results by study are reported in the table in S5 Appendix. (page 16, line 212)
- We have quantified the sparseness of the data and the number of deaths across studies. Additionally, we wish to note for Reviewer #2 that 11 of the 18 studies reporting mortality had 2 or fewer deaths in each arm (page 17, line 212)
- We have described that a meta-analysis for mortality was not conducted due to data sparseness (page 17, line 222).
- We added an additional sentence highlighting that no study’s primary outcome was mortality (page 17, line 224).

This section now reads: “Of the 18 studies reporting mortality, 14 studies had 12 or fewer deaths combined in the intervention and comparator groups. Studies with larger numbers of deaths appeared to have generally similar mortality between trial arms though a formal meta-analysis was not conducted due to sparseness of data. [22, 24, 46, 50]. No study’s primary outcome was mortality.”

With respect to the statement on page 20 (now page 23, line 384) that HbA1c was the only outcome that could be incorporated into the meta-analysis, we have revised this sentence to read: “The only outcome in our review for which a meta-analysis was conducted, HbA1c, is only a surrogate outcome…”

- **Presumably standardised mean differences might be appropriate for HQoL but if the reporting of HQoL makes it hard to synthesise then at least give us the data. It is the only patient reported outcome here and that is what patients are concerned with, their wellbeing, not their glycated haemoglobin.**

Yes, the reporting of quality of life made it difficult to synthesize this outcome. As an authorship team composed of multiple internists and primary care physicians, we strongly agree with Reviewer #2’s sentiment that patients are less concerned with their HbA1c and more concerned about their symptoms and quality of life.

We have clarified that full outcome results by study are reported in the table in S5 Appendix, and we keep a descriptive summary of findings (6 studies showing no differences and 5 studies showing improved quality of life in intervention arm). We have also added a sentence noting that only one study had quality of life as a primary outcome (page 17, lines 226). This section now reads:

“Of the 11 studies reporting quality of life, six studies reported no significant differences [22, 25, 38, 39, 45, 51], and five studies showed improved quality of life in the intervention arm [26, 33, 44, 50, 54]. Seven different scales were used to assess quality of life, and only the Euroqol EQ-5D was implemented more than once [22, 38, 39, 45, 50]. Only one study reported quality of life as a primary outcome [44].”

**Supplementary Appendix S5 The references are very confusing. Table 1 tells us that reference 43 is by Van Olmen which tallies with the bibliography but in Table S5 references 55 to 58 appear alongside that name one of which has him/her as a first author in the bibliography. That is the only one I checked (I was looking to see which low–income country was involved). It would be much better to have a single bibliography or to only cite papers in one of the two given here.**

We can understand why the references appeared confusing. In the body of the main paper, we cite only 1 primary reference for each trial (generally the reference that we perceived to be the study’s main publication). However, many studies had multiple published reports that related to the same trial, and we drew on these other reports when extracting study data.

In Appendix S5 table, we cite each trial’s main publication in the column titled “Study” and also other published reports from the same trial in the column titled “Other reports.” In the example Reviewer #2 brings up, reference 43 (now reference 44) in the main text is the study by Van Olmen, Kegels, Korachais, et. al; in Appendix S5 table, this same citation is numbered reference 55, and other reports from the trial are numbered 56-58.

We would like to provide citations in the appendix all of the published reports that we used in the review. We have clarified the presentation of Appendix S5 by repeating the header across pages.

**Supplementary Appendix S6 Unfortunately using baseline risk as a covariate although appealing may lead to bias. McIntosh (1996); Thompson et al. (1997); Walter (1997) all discuss solutions using various techniques.**

Thank you for these references on the potential bias of using baseline risk as a covariate. We have discussed this issue among the authorship team and, in view of these limitations, we have decided to omit the meta-regression analysis with baseline HbA1c as a covariate.

This decision aligns with our general revision approach of deemphasizing statistical investigation of heterogeneity to make inferential claims. We feel it is most useful to readers to emphasize in the text the descriptive differences in terms of country of interest, type and component of intervention, and risk of bias.

**Supplementary Appendix S8 This does not in fact show the funnel plot overall but is identical to S11 as far as I can see.**

Reviewer #2 is correct; we have corrected Appendix S8 to be the overall funnel plot. We have updated the title in this and other appendices to more clearly denote when the figure refers to all studies or the subset of studies without high risk of bias.

Please note that this appendix is now labeled as Supplementary Appendix S7.

**Supplementary Appendix S9 I think these are for all studies but that could be confirmed in the caption. I do not see much point in presenting a regression type test for the last six of these and suggest omitting it. It would be helpful to provide the x–axis for all the plots. As given here the central estimate for pharmacists does not look in the place I had expected from Figure 2 but that might be an optical effect.**

Yes, this refers to all studies. As mentioned above, we have updated the caption in this and other plots to improve interpretation. In response to Reviewer #2’s suggestion, we have omitted the Egger test results for the last size graphs and added x-axes for all graphs. We also have reviewed the code and double checked all of the funnel plots to ensure that central estimates correspond with the estimates in the forest plots.

(This now refers to Supplementary Appendix S8.)

**Supplementary Appendix S12 Similar comments to S9 but only first one needs regression type test.**

We have incorporated this suggestion.

(This now refers to Supplementary Appendix S1.)

**Points of more substance**

**Coverage**

**When I looked at Appendix S5 my immediate thought was that I had never seen a review on health in LaMICs with so many studies from Malaysia. There are other countries with considerable contributions. Are the authors satisfied that all the studies within any one country are independent? They must share some characteristics in terms of the health system. They might come from the same, or overlapping, research teams. I do not think this needs incorporating in a formal way in a multi–level meta–analysis but some comment would be good.**

We were extremely careful in assessing for duplicate reports or secondary analyses of the same trial. In most cases, we could determine if a publication was part of the same trial by assessing country, intervention details, and, as Reviewer #2 mentions, the authorship team. On at least one occasion (the study by Adibe et. al), we reached out to the study’s corresponding author in order to clarify whether two reports were derived from the same trial.

When we identified multiple reports of the same trial, we added citations in the “Other reports” column of “S5 Appendix: Characteristics of included studies.” We identified multiple reports for 13 of the 38 included studies.

Of the 38 included studies, the three countries most represented were China (n=8), Malaysia (n=5), and India (n=4). We have re-reviewed studies from these countries, and we feel confident that they represent independent trials.

We have added the following sentences to the methods (p. 10, lines 74) to further clarify this issue for readers:

“Multiple reports from the same study were identified by reviewing the country setting, intervention details, and, authorship list. When multiple reports were identified, we linked the reports together for extraction and analysis.”

**Excluding high risk of bias**

**Surely the point is not whether this leads to a move across the arbitrary boundary of statistical significance as page 16 suggests but whether the central estimate is affected to a clinically significant amount. After all if you have fewer studies then all else being equal the confidence interval is going to be wider.**

Given relatively fewer studies within each subgroup, we have opted to omit the reference to statistical significance in the sensitivity analysis of subgroups in the main body of the paper. Interested readers will be able to review the totality of the findings in supplemental appendices S10-11 and make their own interpretations of these data.

**Summary**

**A substantial piece of work with one or two issues to clarify.**

**Michael Dewey**

We thank Reviewer #2 for his thoughtful comments and suggestions. The manuscript is more clear and rigorous because of this review.

**References**

**M J Bradburn, J J Deeks, J A Berlin, and A R Localio. Much ado about**

**nothing: a comparison of the performance of meta–analytical methods**

**with rare events. Statistics in Medicine, 26:53–77, 2007.**

**J Ioannidis, N Patsopoulos, and E Evangelou. Uncertainty in heterogeneity**

**estimates in meta–analyses. British Medical Journal, 335:914–916, 2007.**

**M W McIntosh. The population risk as an explanatory variable in research**

**synthesis of clinical trials. Statistics in Medicine, 15:1713–1728, 1996.**

**S G Thompson, T C Smith, and S J Sharp. Investigating underlying risk**

**as a source of heterogeneity in meta-analysis. Statistics in Medicine, 16:**

**2741–2758, 1997.**

**S D Walter. Variation in baseline risk as an explanation of heterogeneity in**

**meta-analysis. Statistics in Medicine, 16:2883–2900, 1997.**

**-----------------------------------------------------------**

**Reviewer #3:**

**This systematic review and meta-analysis achieves its intended aims and has been conducted with sufficient rigour.**

**The introduction provides a clear rationale of the need for this study.**

We thank Reviewer #3 for these comments.

**It may be of benefit to include example/s of some health service interventions within the introduction (paragraph 2). While the EPOC definition is stated within the methods section, some examples earlier in the introduction would assist readers to interpret the distinction between clinical interventions and health service interventions.**

We have added the following sentence in the introduction on page 7, lines 12-14: “Examples of health systems interventions include quality and safety programs, health information systems, health worker incentives, changes in scope of practice, and many others.”

**The methods section provides sufficient detail as per the PRISMA recommendations. Information is provided with regard to eligible interventions, however, eligible control conditions do not appear to be stated throughout the manuscript. Given the intention of a meta-analysis is to quantify the effects of a specific intervention in relation to a specified control condition, this is an important consideration to include throughout the entire manuscript. This would also assist the reader to determine if the meta-analysis is comparing similar studies. It is possible that the heterogeneity observed may have been due to studies using different control conditions.**

We have added the following sentences to clarify and elucidate this issue:

1. “The comparator group in most studies was usual care as defined by the local program or setting of care. Two studies described the comparator group as enhanced usual care consisting of clinical training for health professionals [23, 24], and in one study the medical fees were waived in the comparator arm [25].” (Results, page 12, line 136)
2. “Both the intervention comparator arm in this trial had out-of-pocked medical fees waived, which may have contributed to catalyzing participants to seek medical care.” (Discussion, page 21, line 336)
3. “Of note, the comparator arm in two of these well-conducted multicomponent clinic-based trials consisted of enhanced usual care [23, 24], potentially causing an attenuation of effect size trial” (Discussion page 20, line 320).

For space reasons, we did not include the comparator description in “S5 Appendix: Characteristics of included studies.” However, our complete data extraction sheet including comparator description will be publicly available to readers in an spreadsheet at our data sharing site:

https://dataverse.harvard.edu/privateurl.xhtml?token=b04cbdc0-7bb2-481f-bbce-e9bc191551c0

**The results section provides a excellent synthesis of both the narrative and quantitative components. When summarizing outcomes, I would find it of interest to state how many of the included studies had a primary outcome of HbA1c (line 195).**

As described in our reponses to Reviewer #2, we have added the following sentence to the summary of outcomes:

“The primary outcome involved changes in HbA1c or the proportion of participants meeting HbA1c goals in 23 studies.” (p. 16, lines 217)

We have also specified the number of studies with primary outcomes of mortality (page 17, line 225), quality of life (page 17, line 231), and cost-effectiveness (page 17, line 237).

**The discussion section solidly interprets the findings in relation to previous literature and acknowledges the strengths and limitations of the review.**

**Overall a well written, well conducted, interesting piece of work.**

We thank Reviewer #3 for these kind comments and the thoughtful review.

**-----------------------------------------------------------**

**Reviewer #4:**

**Dr Flood and coworkers have conducted a systematic review and meta-analysis of reports (trials) on health system intervention for diabetes control in LIMC, using change in HbA1c as main outcome. After extensive searchers they identified 38 eligible studies, of which 34 were included in meta-analysis. They found a significant effect of the intervention with a change in HbA1c of 0.46% overall, 0.37% in multi-component clinic-based interventions, 0.93% in pharmacist task-sharing studies, and -0.27% in trials of diabetes education and support alone. Other pre-specified outcomes were inconsistently reported. They concluded on a likely efficacy of health interventions and advocated for further studies to assess the impact on non-glycaemic outcomes.**

**The review addresses a question of global relevance, the manuscript is generally well written and easy to read. I have the following suggestions to the authors.**

We thank Reviewer #4 for these comments.

1) **There seems to be suggestions that non-English language studies were excluded in the selection process with 20 such studies excluded in the second last stage. Should some of these studies be eligible, then it will be a bias and the authors should rather consider using translated to access the information in non-English language studies for possible inclusion in the review.**

As described in our comments to Reviewer #2, our searches were run without language restrictions in the bibliographic databases. We only included studies that were published in English. To clarify this issue for the reader, we have removed the following sentence referring to the search language (now page 8, line 46): “All searches were run without limits or language restrictions.”

We considered using language translation services. At this point in the review process, we determined that it was not feasible to repeat the screening or full-text review process for non-English language studies. We acknowledge this issue as a limitation on page 23, line 387.

**2) Did the investigators make effort to contact the primary investigators for missing data; there seems to be indications that soem studies were excluded for missing data.**

Yes, as described in our initial submission (page 10, line 82), we contacted all corresponding authors on 2 occasions to obtain outcomes that were not reported or missing, as well as to clarify any questions the review team had during the extraction and analysis. We supplemented our review with unpublished data received from several study authors (who also consented to be name in the Acknowledgements section at the end of the manuscript). We think that obtaining supplemental data is one of the key strengths of the review (as described in page 23, line 397).

2) **The authors have identified possible publication bias from funnel plots and have tried to explore this further using subgroup analyses. However, because of the overall small number of studies included, subgroups analyses are likely to be under powered to reliably explore publication bias. I am advising the authors to consider using the 'trim and fill' methods to impute the missing studies, and see if this results in imputed studies with plausible effect size. Similarly the authors should consider running other sensitivity analyses to confirm the robustness of their findings. I am thinking here of the 'leave one out' approach for instance, to check if some particular studies had more effect on the overall estimates.**

As described above in our responses to Reviewer #2, we have discussed the heterogeneity issue extensively among the authorship team. We have made a general decision to deemphasize statistical investigation of heterogeneity and bias in the paper. We think it is most useful to readers to continue to emphasize in the text how included studies may be different in terms of country of interest, type and component of intervention (i.e., our subgroup analysis), and risk of bias. For example, we have omitted the meta-regression with covariate of baseline HbA1c, and we would prefer to not conduct additional analyses of heterogeneity that were not pre-specified in our protocol. Ultimately, we will defer to Reviewers #2 and #4, as well as the editors, if they deem it necessary to pursue a more detailed investigation of statistical heterogeneity.
