# Supplementary material for: Health system interventions for adults with type 2 diabetes in low- and middle-income countries: A systematic review and meta-analysis
Source: PLoS Med. 2020 Nov 12;17(11):e1003434. doi: 10.1371/journal.pmed.1003434 (PMC7660583; doi:10.1371/journal.pmed.1003434)
Supplement: Attachment — Submitted filename: 2020_09_01 - Responses to reviewers - Flood review.docx [file pmed.1003434.s018.docx]

**Health system interventions for adults with type 2 diabetes in low- and middle-income countries: A systematic review and meta-analysis**

**Responses to Reviewers**

**September 1, 2020**

-----------------------------------------------------------

**Requests from the editors:**

1. **Please address all of the reviewers’ comments below. Notably, we request that you update your study to include non-English studies and the additional sensitivity analyses requested. We appreciate that the inclusion of non-English language studies may require additional expense and effort, but we agree with reviewer #4 that the review would be incomplete without these, particularly given the focus on LMICs.**

Thank you for the opportunity to revise this manuscript.

We have addressed all of the reviewers’ comments below. As we describe in the response to Reviewer #2, the review team has invested a substantial amount of energy and resources to include non-English trials in the review. We also have included the sensitivity analyses requested by Reviewer #4.

2. **Abstract:**
   a. **Please report all of the elements required by PRISMA for abstracts: http://www.plosmedicine.org/article/info:doi/10.1371/journal.pmed.1001419 . Specifically, please include a description of how study quality/risk of bias was assessed, and what the overall quality/risk of bias was found to be.**

We now report all of the elements required by PRISMA for abstracts. With respect to quality/risk of bias, we have added the following text to the abstract:

“GRADE and Cochrane Effective Practice and Organization of Care methods were used to assess risk of bias for the glycemic outcome and to prepare a summary of findings table.” (line 57)

“The certainty of evidence for glycemic control by subgroup was moderate for multicomponent clinic-based interventions but was low or very low for other intervention types” (line 74)

b. **Please give the total number of patients across all included studies.**

We have added the following sentence to the abstract: “There were 21,080 total participants enrolled across included studies and 10,060 total participants in the meta-analysis of HbA1c when accounting for the design effect of cluster RCTs.” (line 61)

c. **Please clarify (here and in the main Methods) that the search dates were “from inception through February 24, 2020”, as appropriate.**

We have clarified this as follows:

In the abstract: “We searched Ovid MEDLINE, Cochrane Library, EMBASE, African Index Medicus, LILACS, and Global Index Medicus from inception of each database through February 24, 2020.” (line 52)

In the manuscript’s methods (last sentence of first paragraph under “Search strategy and selection criteria”): The search dates were from database inception through February 24, 2020. (line 167)

d. **Please include a breakdown of the number of each study type for the included studies (eg. “18 cluster RCTs, 20 individual RCTs”), and regions covered (as from table 1).**

We have included this breakdown:

“Of the 12,921 references identified in searches, we included 39 studies in the narrative review of which 19 were cluster RCTs and 20 were individual RCTs.” (line 59)

e. **Please include line numbers in the margin throughout.**

Line numbers have been added.

3. **Please remove the Competing Interests, Data Availability, Funding and Author Contribution statements – these should be filled in on the online submission form instead. Please keep the Acknowledgements here as they are.**

Thank you; we have removed these sections.

4. **When completing the PRISMA checklist, please use section and paragraph numbers, rather than page numbers. Your checklist also appears to be cut off after item 14 – please include the full checklist.**

We apologize that our previous PRISMA checklist was truncated during the preparation of the appendices. We have revised and included the full checklist with section numbers in the S2 Appendix.

-----------------------------------------------------------

**Reviewer #1: I think that the reviewers have done an adequate job in responding to my initial concerns.**

AU Reply: Thank you.

**The authors conclude that the most robust evidence of benefit is for multi-component interventions. However, this seems at odds with the data in Fig 2. Pharmacist task sharing interventions include more trials (albeit with fewer participants), a larger point estimate for the effect size, and 8/12 trials with individually statistically significant benefits. It appears that the downgrading of the Pharmacist trials is based on the GRADE score in Appendix S12, which is rated as 2/4 for the pharmacy trials compared to 3/4 for the multi-component trials. No discussion is provided on what the actual difference is between the quality scores, though this is central to the main study conclusion. The relevance of this is not only in regard to whether or not the evidence supports pharmacist task sharing interventions, but whether or not single-component interventions in general might be effective. Several other studies in this setting have concluded that multi-component interventions are required to be effective, and the current presentation of this meta-analysis supports this. However, that conclusion turns out to be entirely dependent on a difference in GRADE scores that is not explained or discussed.**

AU REPLY: Thank you for bringing attention to this critical point.

In the Methods section under the Data Analysis subsection, we have clarified the EPOC/GRADE methods used to generate the certainty of evidence assessments:

“We used GRADE and EPOC guidance to assess risk of bias for the glycemic outcome and to prepare a summary of findings table [18-21].” (line 205)

In the Results section (last paragraph before discussion), we also have added a paragraph that discusses in more detail the reasons for downgrading GRADE scores:

“The certainty of evidence using the GRADE/EPOC approach for glycemic control by subgroup was moderate for multicomponent clinic-based interventions but was low or very low for other intervention types (S15 Appendix). The most common reasons for downgrading the certainty of evidence for intervention types were due to concerns regarding risk of bias or inconsistency across studies (S16 Appendix). For example, in the case of pharmacist task-sharing interventions, nine of the 14 studies were classified as being at high risk of bias, five were at unclear risk of bias, and none were at low risk of bias. The absence of high-quality trials resulted in a low certainty of evidence for pharmacist task-sharing studies despite their sizeable pooled HbA1c estimate in the meta-analysis. Conversely, in the case of multicomponent clinic-based interventions, only one of the eight studies were deemed to be at high risk of bias, and three of the studies were at low risk of bias. The result was a moderate certainty of evidence for the glycemic outcome for these interventions despite a lower pooled HbA1c estimate than the pharmacist-led studies.” (line 478)

We have also added as an appendix the EPOC worksheet used to generate the certainty of evidence assessments for the glycemic outcome: “S16 Appendix: Certainty assessment of evidence the glycemic control”

We also wish to note for Reviewer #1 that we have added prediction intervals upon the suggestion of Reviewer #2. In the results, we write:	“The overall HbA1c prediction interval was -1.19% to 0.28%.” (line 407). In the discussion, we synthesize the implications of the prediction intervals as follows:

“In the overall meta-analysis of HbA1c from 35 trials, we found that health system interventions modestly improved glycemic control on average. At the same time, the wide prediction interval overlapping zero in the meta-analysis of HbA1c showed that there was a wide range of effectiveness across studies, and some health systems interventions may not be effective in all settings.” (line 516)

“Further studies in LMICs are needed to assess non-glycemic outcomes and, given the wide prediction intervals, to determine the specific components and details of health system interventions most likely to promote effectiveness and limit potential harms.” (page 596)

Finally, we recognize that the certainty of evidence assessments are subjective and reflect our judgments as reviewers. Yet, our approach is consistent with EPOC methodology and, in fact, now has been strengthened by adding more detail about reasons for downgrading evidence in the added appendix.

-----------------------------------------------------------

**Reviewer #2: The authors have addressed my points but I still have a couple of things to raise.**

**Even is the authors do not wish to do a formal analysis of mortality and the other outcomes I think expecting the reader to work all through S5 is unnecessary when they could be presented by outcome. I would put them in a forest plot but if the authors wish they can suppress the summary estimates.**

AU Reply: Thank you for this suggestion. We agree with Reviewer 2 and have added a forest plot of deaths by study, suppressing the summary estimates. Please see the following appendix: “S6 Appendix: Forest plot of overall deaths by study.”

**I see there is some divergence of opinion between the referees about the heterogeneity and possible small study effects. For the record I have no problem with doing a summary in the presence of extreme statistical heterogeneity as measured by I^2. Whether the clinical heterogeneity precludes it is not for me to say.**

AU Reply: Thank you. We agree that the statistical heterogeneity does not necessarily translate to the clinical nuances and differences across studies. Our approach has been to give the reader all of the quantitative and qualitative data within trials so that a complete view of the evidence can be generated.

**One thing I should have suggested and I apologise for not having done this at the beginning is that prediction intervals in addition to confidence intervals would be useful. They give a picture of where the next study would be likely to fall which I would imagine is more useful to someone working in public health in a different country than knowing the properties of the distribution of the summary statistic. Riley et al have argued for the use of prediction intervals https://doi.org/10.1136/bmj.d549 and it is not super-difficult to calculate them even if the authors' software does not do it.**

AU Reply: We thank the reviewer for this excellent suggestion. We have added prediction intervals to the main forest plot as recommended.

We describe the reasons for adding them in the methods:

“To provide a range of the effects of individual studies, we calculated an overall prediction interval [24].” (line 224)

In the results, we write:

“The overall HbA1c prediction interval was -1.19% to 0.28%.” (line 410)

In the discussion, we synthesize the implications of the prediction intervals as follows:

“In the overall meta-analysis of HbA1c from 35 trials, we found that health system interventions modestly improved glycemic control on average. At the same time, the wide prediction interval overlapping zero in the meta-analysis of HbA1c showed that there was a wide range of effectiveness across studies, and some health systems interventions may not be effective in all settings.” (line 516)

“Further studies in LMICs are needed to assess non-glycemic outcomes and, given the wide prediction intervals, to determine the specific components and details of health system interventions most likely to promote effectiveness and limit potential harms.” (page 596)

**In the presence of the heterogeneity I do not think it is worth spending much time on possible small study effects and trim and fill has rather fallen out of favour recently.**

AU Reply: Thank you for your comment. While we agree with you on this point, we are also sensitive to the fact that debate regarding the impact of clinical and statistical heterogeneity remains. Therefore, honoring the suggestion of Reviewer 4 and the editors, we have added additional analysis using the trim-and-fill method and the leave-one-out method. Please see our response to Reviewer 4 for additional details and explanation.

**I think I failed to explain adequately my concern about lack of independence of studies within a country. It is not just a concern about the same research team being involved which I am sure the authors have checked but also the fact that studies in a single country must share the same background health system and so will be more similar than those from a different country. I do not think country needs inclusion in a formal analysis but I would mention this as a possible limitation.**

AU Reply: This is an important limitation; thank you for pointing it out. We have added the following sentence to the paragraph on limitations in the Discussion:

“Multiple trials were included within some countries, but we did not formally account for a potential lack of independence among studies conducted within the same health system context. This aspect reflects a limitation of the evidence generated rather than one of the analysis itself.” (line 617)

**Michael Dewey**

-----------------------------------------------------------

**Reviewer #4: Thanks for the opportunity of reading the revised manuscript. I note however that the authors have basically declined answering my two major comments:**

AU Reply: Thank you for these comments and your review. We apologize if your view is that we did not answer your two queries. Below, we specifically describe how we have addressed your two major comments in this revision:

1) **They seem to be unable to access appropriate translation service to enable them assessing all potentially eligible studies; and therefore have limited inclusion to only studies published in English. In doing so, they have excluded at least 20 studies from further assessment due to language issue. Not only this deliberately introduces publication bias, but I am not sure if it would be acceptable for robust systematic review. The authors should consider the option of accounting at least for few other major international languages.**

AU Reply: We have enhanced the review to include non-English language studies (which were listed but not formally included in the prior iteration). Consequently, in this revision, there are no studies excluded for language reasons. We appreciate that this suggestion has strengthened our review.

In the methods, we describe our approach to language inclusion:

“We used language proficiency among members of our review team and Google Translate to abstract data from non-English trials [16].” (line 194)

Multiple study authors including the lead author are proficient in Spanish, which also facilitated language inclusions. Additionally, the citation below references a recent paper supporting the use of Google Translate for abstraction of data from clinical trials in systematic reviews:

Jackson JL, Kuriyama A, Anton A, Choi A, Fournier JP, Geier AK, et al. The Accuracy of Google Translate for Abstracting Data From Non-English-Language Trials for Systematic Reviews. Ann Intern Med. 2019; 171(9):677-9. http://dx.doi.org/10.7326/m19-0891 PMID: 31357212

Through these approaches, we added three new studies to the analysis, two of which were separate reports of trials with English versions that had been previously included. In the meta-analysis in this revision, there were modest differences in the overall HbA1c estimate across the 35 trials now included in the meta-analysis of HbA1c, and statistical significance persisted. The estimate for the subgroup of pharmacist-led interventions changed slightly from -0.93% (-1.29 to -0.57, I^2^ 91.7) in the previous version to -0.87% (-1.20 to -0.53, I^2^ 91.0) in the current manuscript.

2) **I proposed that they use sensitivity analyses (trim and fill) to explore the effect of missing studies imputation on the publication bias. I am not convinced by the rebuttals of the authors. Should the sensitivity analyses show that imputed studies have plausible effect sizes, then the reader needs to know, since accounting for missing studies could completely change the overall estimates from the analyses conducted by the authors. I also advised the authors to run other sensitivity analyses (leave one out) to confirm the robustness of their findings, what they haven't done.**

AU Reply: We appreciate this suggestion and initially declined from doing this given that (1) there were differences in opinion among the reviewers (including the statistical reviewer) on the best way forward, and (2) the general discourse among review methodologists that statistical tools represent imperfect approaches to addressing publication bias.

In our study, given how the evidence was highly variable in terms of effect size and risk of bias, we initially were concerned that adding statistical tests for bias might lead to a false sense of security among readers. Below are articles that have influenced our thoughts on the topic of statistical investigation of publication bias using funnel plots:

Schmid CH. Discussion of "quantifying publication bias in meta-analysis" by Lin et al. Biometrics 2018; 74(3): 797-9.

Lin L, Chu H. Quantifying publication bias in meta-analysis. Biometrics 2018; 74(3): 785-94.

Lau J, Ioannidis JP, Terrin N, Schmid CH, Olkin I. The case of the misleading funnel plot. BMJ 2006; 333(7568): 597-600.

However, at this stage (especially given the addition of new studies), we acknowledge that adding formal analyses can help reassure readers about this aspect and thus have added both of these analyses. Overall, we find that these additional sensitivity analyses do not change the statistical significance of the overall average HbA1c estimates compared to the primary analysis, though the trim-and-fill method suggests that from a statistical perspective there could be multiple missing studies that would reduce the overall effect size.

The full results of these analyses are presented as figures in the following new supplementary appendices:

S10 Appendix: Funnel plot generated using trim-and-fill method

S14 Appendix: Forest plot generated using leave-one-out method

In the methods of the manuscript, we add the following descriptions:

“[W]e assessed the influence of individuals studies by using the leave-one-out method to recalculate estimates omitting one study at a time ^4^.” (line 227)

“The trim-and-fill method was also applied to impute the number of studies potentially missing from the meta-analysis and to re-estimate an overall effect size accounting for publication bias [27].” (line 231)

In the results section, we add the following text:

“Using the trim-and-fill method, we estimated that there were eight missing studies, and inclusion of these imputed studies resulted in an estimated overall HbA1c mean difference of -0.28% (95% CI: -0.43 to -0.13, S10 Appendix)…. In the second sensitivity analysis using the leave-one-out method, we found that exclusion of the study with the largest effect size[42] would result in a HbA1c mean difference and heterogeneity of -0.39% (95% CI: -0.52 to -0.26, I^2^ 84.5%, S14 Appendix).” (line 468)

In the discussion, we write:

“There was statistical evidence for publication bias and substantial differences in the quality of underlying studies that limited the certainty of evidence of glycemic benefit for intervention types” (line 607)
